# Identifying Relationships among Genomic Disease Regions: Predicting Genes at Pathogenic SNP Associations and Rare Deletions

**DOI:** 10.1371/journal.pgen.1000534

**Published:** 2009-06-26

**Authors:** Soumya Raychaudhuri, Robert M. Plenge, Elizabeth J. Rossin, Aylwin C. Y. Ng, International Schizophrenia Consortium, Shaun M. Purcell, Pamela Sklar, Edward M. Scolnick, Ramnik J. Xavier, David Altshuler, Mark J. Daly

**Affiliations:** 1 Program in Medical and Population Genetics, Broad Institute of MIT and Harvard, Cambridge, Massachusetts, United States of America; 2 Center for Human Genetic Research, Massachusetts General Hospital, Boston, Massachusetts, United States of America; 3 Division of Rheumatology, Immunology and Allergy, Brigham and Women's Hospital, Harvard Medical School, Boston, Massachusetts, United States of America; 4 Harvard Medical School – Partners HealthCare Center for Genetics and Genomics, Boston, Massachusetts, United States of America; 5 Harvard-MIT Health Sciences and Technology, Cambridge, Massachusetts, United States of America; 6 Center for Computational and Integrative Biology, Massachusetts General Hospital, Boston, Massachusetts, United States of America; 7 Gastroenterology Unit, Massachusetts General Hospital, Boston, Massachusetts, United States of America; 8 Stanley Center for Psychiatric Research, Broad Institute of MIT and Harvard, Cambridge, Massachusetts, United States of America; 9 Psychiatric and Neurodevelopmental Genetics Unit, Massachusetts General Hospital, Boston, Massachusetts, United States of America; 10 Department of Psychiatry, Massachusetts General Hospital, Boston, Massachusetts, United States of America; 11 Department of Molecular Biology, Massachusetts General Hospital, Boston, Massachusetts, United States of America; 12 Department of Genetics, Harvard Medical School, Boston, Massachusetts, United States of America; 13 Diabetes Unit, Massachusetts General Hospital, Boston, Massachusetts, United States of America; Princeton University, United States of America

## Abstract

Translating a set of disease regions into insight about pathogenic mechanisms requires not only the ability to identify the key disease genes within them, but also the biological relationships among those key genes. Here we describe a statistical method, Gene Relationships Among Implicated Loci (GRAIL), that takes a list of disease regions and automatically assesses the degree of relatedness of implicated genes using 250,000 PubMed abstracts. We first evaluated GRAIL by assessing its ability to identify subsets of highly related genes in common pathways from validated lipid and height SNP associations from recent genome-wide studies. We then tested GRAIL, by assessing its ability to separate true disease regions from many false positive disease regions in two separate practical applications in human genetics. First, we took 74 nominally associated Crohn's disease SNPs and applied GRAIL to identify a subset of 13 SNPs with highly related genes. Of these, ten convincingly validated in follow-up genotyping; genotyping results for the remaining three were inconclusive. Next, we applied GRAIL to 165 rare deletion events seen in schizophrenia cases (less than one-third of which are contributing to disease risk). We demonstrate that GRAIL is able to identify a subset of 16 deletions containing highly related genes; many of these genes are expressed in the central nervous system and play a role in neuronal synapses. GRAIL offers a statistically robust approach to identifying functionally related genes from across multiple disease regions—that likely represent key disease pathways. An online version of this method is available for public use (http://www.broad.mit.edu/mpg/grail/).

## Author Summary

Modern genetic studies, including genome-wide surveys for disease-associated loci and copy number variation, provide a list of critical genomic regions that play an important role in predisposition to disease. Using these regions to understand disease pathogenesis requires the ability to first distinguish causal genes from other nearby genes spuriously contained within these regions. To do this we must identify the key pathways suggested by those causal genes. In this manuscript we describe a statistical approach, Gene Relationships Across Implicated Loci (GRAIL), to achieve this task. It starts with genomic regions and identifies related subsets of genes involved in similar biological processes—these genes highlight the likely causal genes and the key pathways. GRAIL uses abstracts from the entirety of the published scientific literature about the genes to look for potential relationships between genes. We apply GRAIL to four very different phenotypes. In each case we identify a subset of highly related genes; in cases where false positive regions are present, GRAIL is able to separate out likely true positives. GRAIL therefore offers the potential to translate disease genomic regions from unbiased genomic surveys into the key processes that may be critical to the disease.

## Introduction

An emerging challenge in genomics is the ability to examine multiple disease regions within the human genome, and to recognize a subset of key genes that are involved in a common cellular process or pathway. This is a key task to translate experimentally ascertained disease regions into meaningful understanding about pathogenesis. The importance of this challenge has been highlighted by advances in human genetics that are facilitating the rapid discovery of disease regions in the form of genomic regions around associated SNPs (single nucleotide polymorphisms) [1]–[6] or CNVs (copy number variants) [7]–[10]. These disease regions often overlap multiple genes – though only one is typically relevant to pathogenesis and the remaining are spuriously implicated by proximity. The difficulty of this task is heightened by the limited state of cataloged interactions, pathways, and functions for the vast majority of genes. However, undefined gene relationships might often be conjectured from the literature, even if they are not explicitly described yet.

The general strategy of using function to prioritize genes in disease regions has been substantially explored [11]–[18]. However, predicted disease genes have not, in general, been easily validated. Thus far, published approaches have utilized a range of codified gene information including protein-interaction maps, gene expression data, carefully constructed gene networks based on multiple information sources, predefined gene sets and pathways, and disease-related keywords. We propose, instead, to use a flexible metric of gene relatedness that not only captures clearly established close gene relationships, but also has the ability to capture potential undocumented or distant ones. Such a metric may be a more powerful tool to approach this problem rather then relying on incomplete databases of gene functions, interactions, or relationships.

To this end, we use established statistical text mining approaches to quantify *relatedness* between two genes – specifically, gene *relatedness* is the degree of similarity in the text describing them within article abstracts. The published literature represented in online PubMed abstracts encapsulates years of research on biological mechanisms. We and others have shown the great utility of statistical text mining to rapidly obtain functional information about genes, including protein-protein interactions, gene function annotation, and measuring gene-gene similarity [19]–[22]. Text is an abundant and underutilized resource in human genetics, and currently a total of 140,000 abstracts from articles that reference human genes are available through PubMed [23]. Additional valuable information can be seamlessly gained by including more than 100,000 references from orthologous genes; many important pathways have been more thoroughly explored in model systems than in humans.

We have developed a novel statistical method to evaluate the degree of relatedness among genes within disease regions: *Gene Relationships Among Implicated Loci* (GRAIL). Given only a collection of disease regions, GRAIL uses our text-based definition of relatedness (or alternative metrics of relatedness) to identify a subset of genes, more highly related than by chance; it also assigns a select set of keywords that suggest putative biological pathways. It uses no information about the phenotype, such as known pathways or genes, and is therefore not tethered to potentially biased pre-existing concepts about the disease.

In addition to a flexible text-based metric of relatedness, GRAIL's ability to successfully connect genes also leverages a statistical framework that carefully accounts for differential gene content across regions. We assume that each region contains a single pathogenic gene; therefore narrow regions with one or just a few genes are more informative than expansive regions with many genes, since they are likely to have many irrelevant genes. To take advantage of this, we have designed GRAIL to set a lower threshold in considering relatedness for those genes in narrow regions, allowing for more distant relationships to be considered; on the other hand it sets a more stringent threshold for genes located in expansive mutligenic regions and considers only the very closest of relationships. This strategy prevents large regions with many genes from dominating the analysis.

In this paper we apply GRAIL to four phenotypes. In each case GRAIL is able to identify a subsets of genes enriched for relatedness – more than expected by random chance. We demonstrate enrichment for relatedness among true disease regions rigorously based on both GRAIL's theoretically derived *p*-value and also based on parallel analysis of either (1) carefully selected random regions matched for gene content and size or (2) experimentally derived false positive disease regions.

GRAIL is able to identify subsets of highly related genes among validated SNP associations. First we use GRAIL to identify related genes from SNPs associated with serum lipid levels; GRAIL correctly identifies genes already known to influence lipid levels within the cholesterol biosynthesis pathway. In comparison to randomly selected matched SNP sets, the set of lipid SNPs demonstrate significantly more relatedness. Second, we use GRAIL to identify significantly related genes near height-associated SNPs; these genes highlight plausible pathways involved in height. In comparison to randomly selected matched SNP sets, the set of height SNPs also demonstrate significantly more relatedness.

Encouraged by GRAIL's ability to recognize biologically meaningful connections, we tested its ability to distinguish true disease regions from false positive regions in two practical applications in human genetics. First, in Crohn's disease, we start with a long list of putative SNP associations from a recent GWA (genome-wide association) meta-analysis [24]. We demonstrate that a substantial fraction of these SNPs contain highly related genes—far beyond what can be expected by chance. We demonstrate that many of these SNPs subsequently validate in an independent replication genotyping experiment. Second, in schizophrenia, we previously identified an over-representation of rare deletions in schizophrenia cases compared to controls [8]. Despite the statistical excess, it is challenging to identify exactly which case deletions are causal, given the relatively high background rate of rare deletions in controls. Using GRAIL however, we are able to demonstrate that a subset of case deletions contain related genes. We further demonstrate that these genes are highly and significantly enriched for central nervous system (CNS) expressed genes. In stark contrast, GRAIL finds no excess relatedness among genes implicated by case deletions.

## Results

### Summary of statistical approach

GRAIL relies on two key methods: (1) a novel statistical framework that assesses the significance of relatedness between genes in disease regions (2) a text-based similarity measure that scores two genes for relatedness to each other based on text in PubMed abstracts. Details for both are presented in the Methods.

**Figure 1:**
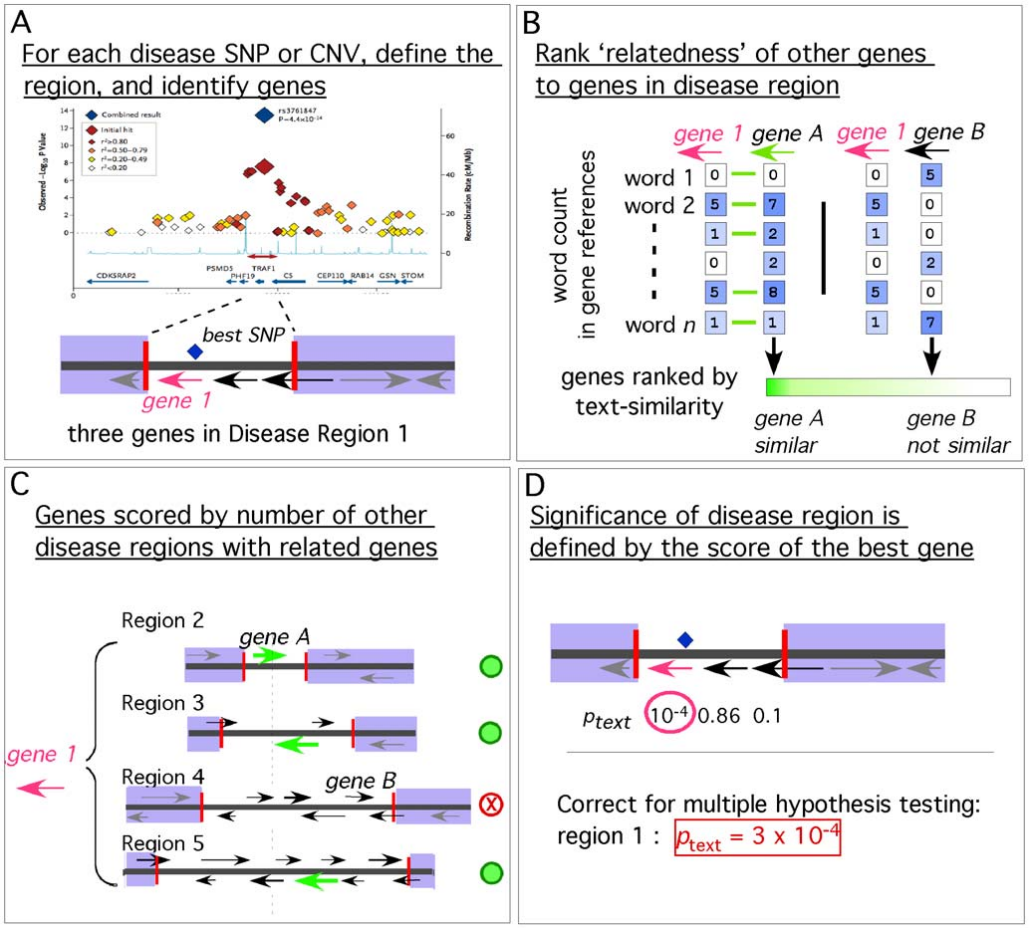
Gene Relationships Among Implicated Loci (GRAIL) method consists of four steps. (A) Identifying genes in disease regions. For each independent associated SNP or CNV from a GWA study, GRAIL defines a disease region; then GRAIL identifies genes overlapping the region. In this region there are three genes. We use *gene 1* (pink arrow) as an example. (B) Assess relatedness to other human genes. GRAIL scores each gene contained in a disease region for relatedness to all other human genes. GRAIL determines gene relatedness by looking at words in gene references; related genes are defined as those whose abstract references use similar words. Here *gene 1* has word counts that are highly similar to *gene A* but not to *gene B*. All human genes are ranked according to text-based similarity (green bar), and the most similar genes are considered related. (C) Counting regions with similar genes. For each gene in a disease region, GRAIL assesses whether other independent disease regions contain highly significant genes. GRAIL assigns a significance score to the count. In this illustration *gene 1* is similar to genes in three of the regions (green arrows), including gene *A*. (D) Assigning a significance score to a disease region. After all of the genes within a region are scored, GRAIL identifies the most significant gene as the likely candidate. GRAIL corrects its significance score for multiple hypothesis testing (by adjusting for the number of genes in the region), to assign a significance score to the region.

The GRAIL statistical framework consists of four steps (see Figure 1). First, given a set of disease regions we identify the genes overlapping them (Figure 1A); for SNPs we use LD (linkage disequilibrium) characteristics to define the region. Second, for each overlapping gene we score all other human genes by their relatedness to it (Figure 1B). In this paper we use a text-based similarity measure; alternative measures of relatedness, for example similarity in gene annotations or expression data, could be easily applied instead [25],[26]. Third, for each gene we count the number of independent regions with at least one highly related gene (Figure 1C); here the threshold for relatedness varies between regions depending on the number of genes within them. We assign a *p*-value to that count. Fourth, for each disease region we select the single most connected gene as the key gene. We assign the disease region that key gene's *p*-value after adjusting for multiple hypothesis testing (if there are multiple genes within the region) (Figure 1D). This final score is listed in this paper as *p_metric_* where the *metric* is *text*, *expression*, or *annotation* based. Very low *p_text_* scores for one region indicate that a gene within it is more related to genes in other disease regions through PubMed abstracts than expected by chance. Simulations on random groups of SNPs demonstrate that the *p_text_* values approximately estimate Type I error rates, being approximately uniformly distributed under the null hypothesis (see Figure S1). However, we recommend the use of careful simulations or controls rather than actual theoretical *p*-values to reinforce the significance of GRAIL's findings – as we do in the examples below.

The text-based similarity metric is based on standard approaches used in statistical text mining. To avoid publications that report on or are influenced by disease regions discovered in the recent scans, we use only those PubMed abstracts published prior to December 2006, before the recent onslaught of GWA papers identifying novel associations. This approach effectively avoids the evaluation of gene relationships being confounded by papers listing genes in regions discovered as associated to these phenotypes. In addition to including primary abstract references about genes listed in Entrez Gene, we augment our text compendium with references to orthologous genes listed in Homologene [23]; this increases the number of articles available per gene from 6 to 12 (see Table 1). We note that the distribution of articles per gene is skewed toward a small number of genes with many references; 0.4% of genes are referenced by >500 articles, while 26% of genes are referenced by <5. In fact 2,034 genes could not be connected to any abstracts at all. For each abstract we convert free text into vectors of word counts [19]. For each gene we define a word vector that consists of averaged word counts from document references to it. Pairwise gene relatedness is then the correlation between the vectors of word counts between two genes. Two genes that are referenced by abstracts using the same sorts of words will receive a high similarity score, whereas two genes that have abstract references that largely use a different vocabulary will receive a low score (Figure 1B). Importantly, genes do not need to be co-cited in the same document to be identified as highly similar.

**Table 1:** Text resources.

				Refs/gene			
	Genes	Articles	References	mean	median	mean	median
Standard	18,690	137,395	260,658	13.9	6	1.9	1
Homologs	15,990	138,720	434,690	27.1	13	3.1	1
Combined	18,875	259,638	599,537	31.7	12	2.3	1

We obtained text from abstracts relevant to human genes from PubMed on December 2006. In the first row we list the number of human genes with any references listed, the total number of abstracts referencing them, and the total number of gene references. We then list the mean and median number of abstract references per gene, and also the mean and median number of gene references per abstract. We used Homologene to identify human gene orthologs, and obtained text for those genes; information about those genes and references is listed in the second row. We combined the two pools of gene references to create a large combined database of 18,875 genes with 599,537 references to 259,638 articles, described in the third row.

After regions are scored with GRAIL, PubMed text can be used to identify keywords that may provide insight into the underlying biological pathways. We define these keywords as those words that most strongly link the significant genes in each region, that is the words with overall greatest weight across all of the text vectors from those genes.

Since the GRAIL framework can be easily used with any gene relatedness metric, we also devised and tested two alternative metrics derived from Gene Ontology (GO) annotations [27] and an mRNA expression atlas consisting of expression measurements across multiple human tissues (The Novartis Gene Expression Atlas) [28]. These metrics are described in greater detail in Methods.

### Evaluating relationships between known associated SNPs: lipid levels and height

**Figure 2:**
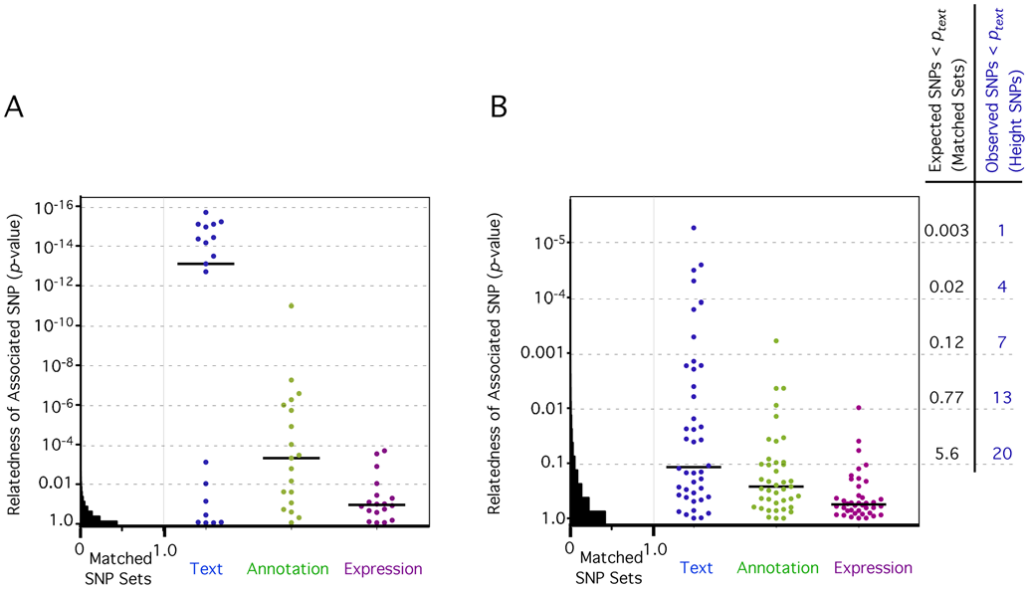
SNPs associated with lipid metabolism and height contain genes related to each other. (A) 19 SNPs associated with lipid metabolism. The *y*-axis plots the *p_text_* values on a log scale, with increasing significance at the top. The histogram on the left side of the graph illustrates values for matched SNP sets. 88.6% of those SNPs have *p_text_* values that are >0.1. The scatter plot on the right illustrates *p_text_* values for actual serum cholesterol associated SNPs (blue dots). Black horizontal line marks the median *p_text_* value. We assessed the same SNP with similarity metrics based on gene annotation (green dots) and gene expression correlation (purple dots). (B) 42 SNPs associated with height. Similar plot for 42 height associated SNPs. The histogram on the left of the graph illustrates *p_text_* values for random SNP sets carefully matched to height-associated SNP set. 86.5% of those SNPs have *p_text_* values that are >0.1. The scatter plot on the right illustrates *p_text_* values for actual SNPs associated with height (blue dots). Black horizontal line marks the median *p_text_* value. We assessed the same SNP with similarity metrics based on gene annotation (green dots) and gene expression correlation (purple dots). On the right we list for each *p_text_* threshold the number of *expected* SNPs less than the threshold based on matched sets, and the number of *observed* SNPs less than the threshold among height associated SNPs.

We first applied GRAIL to a set of 19 validated SNPs associated with triglyceride, LDL, and/or HDL levels [5],[6]. Since 14 SNPs (out of 19) are near genes that are known members of lipid metabolism pathways, we hypothesized that GRAIL should be able to identify these genes accurately. A total of 87 genes were implicated by the 19 associated SNPs. Of the 14 SNPs near compelling candidate genes, 13 obtained *p_text_* scores<0.01 (Figure 2A, Table S1). GRAIL correctly identified those genes implicated in lipid metabolism from each of these 14 regions. To asses the significance of these findings, we applied GRAIL to 1000 random matched SNP sets; each set consisted of 19 SNPs randomly selected from a commercial genotyping array which implicated a similar total of 87±10 genes. In contrast to lipid associated SNPs, not a single matched random set contained 13 SNPs that obtained *p_text_* scores≤0.01; on average matched sets had 0.26 (maximum 6) SNPs with *p_text_*≤0.01 (Figure 2A). Thus, there is substantial enrichment for highly connected genes captured by true lipid associated SNPs.

Despite relatively comprehensive lipid biology annotation, GO does not identify relationships between regions as effectively as published text (Figure 2A). A total of 12 out of the 19 associated SNPs obtained *p_annotation_*<0.01. Relationships between highest scoring candidate genes are explained by several shared GO codes including: *GO:0008203* (‘cholesterol metabolic process’), *GO:0016125* (‘sterol metabolic process’), *GO:0006629* (‘lipid metabolic process’), *GO:0008202* (‘steroid metabolism’), and *GO:0005319* (‘lipid transporter activity’). Gene expression does not identify relationships between regions as effectively as text, either (Figure 2A). A subset of 4 associated SNPs obtain *p_expression_*<0.01. The regions with the most significantly connected genes have similar tissue-specific expression profiles. The highest expression is in four samples taken from adult and fetal liver tissues, known to play a major role in cholesterol metabolism. While associated SNPs are less connected with these alternative metrics, they do seem to leverage the appropriate functional variables and provide valuable phenotypic information.

We next applied GRAIL to 42 validated SNP associations to adult height in recent GWA studies [2]–[4]. This application tests GRAIL's ability to connect genes in the absence of functional literature connecting the phenotype to the relevant pathways. In contrast to lipid metabolism, all associated common SNPs were identified in 2007 and 2008 and the underlying biological pathways involved in height are still poorly understood. This insures independence between association results and the functional literature from before 2007 that is mined in this study. In most cases the key genes are not yet known.

The 42 height SNPs implicated a total of 185 genes (Table S2). Of these 42 regions, 13 obtained *p_text_* scores<0.01 (Figure 2B). For comparison, we used GRAIL to score 1000 matched SNP sets; as before each set consisted of 42 SNPs randomly selected from a commercial array and implicated a total of 185±10 genes. Not a single random set contained 13 SNPs that obtained *p_text_* scores≤0.01; on average matched sets had 0.77 (maximum 10) SNPs with *p_text_* scores<0.01. Thus, we present clear statistical evidence that GRAIL identifies genes with non-random functional connections among associated loci.

Strikingly, the top five keywords linking the genes were ‘hedgehog’, ‘histone’, ‘bone’, ‘cartilage’, and ‘growth’ (see Table S3 for a more complete list). Of note, ‘height’, does not emerge as a keyword since these genes had not been previously related to height. For comparison, the top five keywords for lipid metabolism associated SNPs were ‘lipoprotein’, ‘cholesterol’, ‘lipase’, ‘apolipoprotein’, and ‘triglyceride’ (Table S3). These results are particularly noteworthy as this analysis uses only a simple list of SNPs implicated by GWA studies—no specific biological pathways or mechanisms or phenotype details are assumed.

### Genetic associations to Crohn's disease and schizophrenia—predicting disease regions

After successfully applying GRAIL to validated associations for two phenotypes, we hypothesized that GRAIL could also be used to prospectively identify true disease regions, based on the relatedness of genes within them, from false positive regions. We tested GRAIL's ability to distinguish disease regions from a longer list of results containing a large number of false positive regions as well in two separate human genetics applications.

A recent GWA meta-analysis in Crohn's disease identified 74 independent SNPs as nominally significant (*p*<5×10^−5^) [24]. While the excess beyond chance suggested many of these regions were likely true positives, up to half of these regions should by necessity be unrelated to Crohn's and simply represent the tail of the null distribution. Thus we sought to explore whether GRAIL could identify a subset of these SNPs that implicate an inter-connected set of genes, and whether those represented true associations that could be validated.

In a now published replication genotyping of the 74 SNPs, 30 replicated convincingly when tested in independent samples (defined as having one-tailed association *p*-values<0.0007 in replication samples and two tailed association *p*-values<5×10^−8^ overall), confirming true positive associations, whereas 22 convincingly failed to replicate (defined as overall association *p*-value rising to >10^−4^); the remaining 22 regions had intermediate levels of significance following replication (and can be considered as yet unresolved associations) [24].

We applied GRAIL prospectively to these 74 nominally associated SNPs. GRAIL was initially operated independent of any knowledge of the contemporaneous replication genotyping experiment. Each region contained between 1 and 34 genes, except for two regions that contained no genes and were not scored. GRAIL identified 13 regions as significant (achieving *p_text_* scores<0.01), as with the previous examples far in excess of chance.

**Figure 3:**
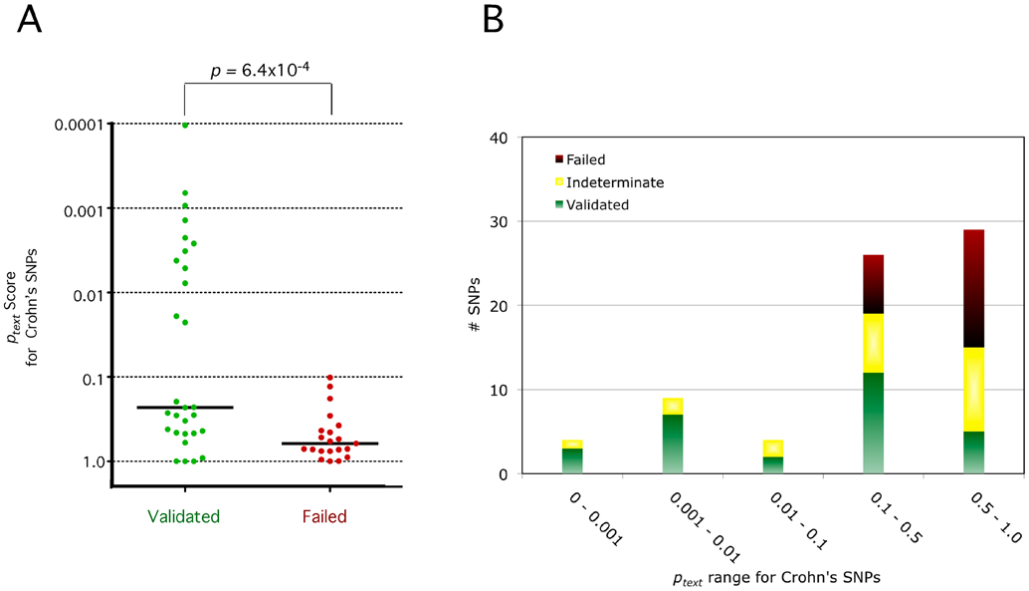
GRAIL predicts Crohn's disease SNPs. (A) Validated versus Failed SNPs. Prior to replication, GRAIL scored Crohn's SNPs that emerged from a meta-analysis study. Results from follow-up testing either validated Crohn's SNPs, or identified those SNPs that failed. We produce a scatter plot of the significance of text-based similiarty (*p_text_*) for validated regions (green) versus regions that failed to replicate (red). Black horizontal lines mark the median *p_text_* values. The distribution of scores for failed SNPs resembles a random distribution of *p*-values. The distribution of scores for validated SNPs is significantly different; almost ½ of these SNPs obtain *p_text_* scores<0.1. (B) Histogram of text-based scores for Crohn's disease candidate regions. Here we plot a histogram of *p*_text_ scores for 74 Crohn's disease SNPs. Validated SNPs (green) have *p*_text_ values that are enriched for significant values. Indeterminate SNPs (yellow) have a subset of *p*_text_ values that are significant. Failed SNPs (Red) have all of their *p*_text_ scores>0.1.

Of those 13 regions, 10 were among the set that convincingly validated in subsequent replication (Table 2)—the remaining three had indeterminate levels of significance. By contrast, only 20 of 63 SNPs remaining SNPs validated (Table S4). Disease regions that replicate have more significant GRAIL scores than those that failed (*p* = 0.00064, one-tailed rank-sum test, Figure 3A). As with randomly selected SNP lists, the distribution of scores for the 21 failed regions was indistinguishable from a random (uniform) distribution of *p*-values (Figure 3B).

**Table 2:** High scoring regions from a Crohn's disease GWA meta-analysis.

SNP	Chr	Position (HG17)	*p_association_*	Replication Study Result	N (genes)	Implicated Gene	*p*_text_
rs2066845	16	49314041	1.5E-24	VALIDATED	3	*NOD2*	0.00010
rs10863202	16	84545499	1.4E-05	INDETERMINATE	4	*IRF8*	0.00058
rs10045431	5	158747111	1.9E-13	VALIDATED-NOVEL	1	*IL12B*	0.00066
rs11465804	1	67414547	3.3E-63	VALIDATED	1	*IL23R*	0.00094
rs2476601	1	114089610	7.3E-09	VALIDATED-NOVEL	8	*PTPN22*	0.0014
rs762421	21	44439989	7.0E-10	VALIDATED-NOVEL	1	*ICOSLG*	0.0023
rs2188962	5	131798704	1.2E-18	VALIDATED	9	*IRF1*	0.0026
rs917997	2	102529086	1.1E-05	INDETERMINATE	5	*IL18RAP*	0.0027
rs11747270	5	150239060	1.7E-16	VALIDATED	3	*IRGM*	0.0032
rs2738758	20	61820069	2.7E-06	INDETERMINATE	10	*TNFRSF6B*	0.0038
rs9286879	1	169593891	7.7E-10	VALIDATED-NOVEL	4	*TNFSF18*	0.0042
rs2301436	6	167408399	5.2E-13	VALIDATED-NOVEL	3	*CCR6*	0.0052
rs4263839	9	114645994	1.3E-10	VALIDATED	2	*TNFSF8*	0.008
rs3828309	2	233962410	1.2E-32	VALIDATED	4	*USP40*	0.019
rs744166	17	37767727	3.4E-12	VALIDATED-NOVEL	2	*STAT3*	0.023
rs7758080	6	149618772	4.4E-06	INDETERMINATE	4	*SUMO4*	0.033
rs7161377	14	75071147	2.3E-05	INDETERMINATE	1	*BATF*	0.09

Here we list a subset of the 74 regions that emerged from a Crohn's disease GWA meta-analysis that GRAIL assigned the most compelling *p_text_* scores to. The first three columns list information about the associated SNP. The fourth column lists the combined *p*-value of association from a GWA meta-analysis and subsequent replication. The fifth column indicates whether the region was validated, indeterminate, or failed in replication. Those regions that represent novel findings, not previously published are also indicated. The sixth column lists the number of genes in the disease region, and the seventh column lists the candidate gene identified by GRAIL. The eighth column lists the regions *p_text_* score.

Using these Crohn's results, we have compared GRAIL's performance to four other competing algorithms that also use functional information to prioritize genes, and GRAIL's performance is superior at predicting true positive associations (see Text S1, Figure S2, Table S5, Table S6).

As a further test of GRAIL, we then evaluated the next most significant 74 associated SNPs that emerged from the Crohn's disease GWA meta-analysis (association *p*-values ranging from 5×10^−5^ to 2×10^−4^). Out of the 75 regions, 8 are not near any gene, and we did not score them. The remaining 67 regions were tested with GRAIL for relationships to the 52 replicated and indeterminate regions that emerged following replication. Two emerge with highly significant GRAIL scores: rs8178556 on chromosome 21 (*IFNAR1*, *p_text_* = 1.7×10^−4^) and rs12928822 on chromosome 16 (*SOCS1*, *p_text_* = 8.2×10^−4^) suggesting these independent regions may lead to novel associated SNPs for Crohn's disease (see Table S7).

We next applied GRAIL to recently published sets of rare deletions seen in schizophrenia cases and matched controls. Multiple groups have recently demonstrated that extremely rare deletions, many of which are likely de novo, are notably enriched in schizophrenia [8]–[10],[29]. However, since rare deletions occur frequently in healthy individuals as well, many of these case deletions will also be non-pathogenic. In fact, we previously found that large (>100 kb), gene overlapping, singleton, deletions were present in 4.9% of cases but also in 3.8% of controls, suggesting that over two-thirds of these deletions are not relevant to disease [8]. We identified 165 published de-novo or case-only deletions of >100 kb overlapping at least one gene; a total of 511 genes are deleted or disrupted by these deletions [8],[9],[10]. Additionally, we identified 122 regions similar control-only deletions; a total of 252 genes are deleted or disrupted by these deletions.

**Figure 4:**
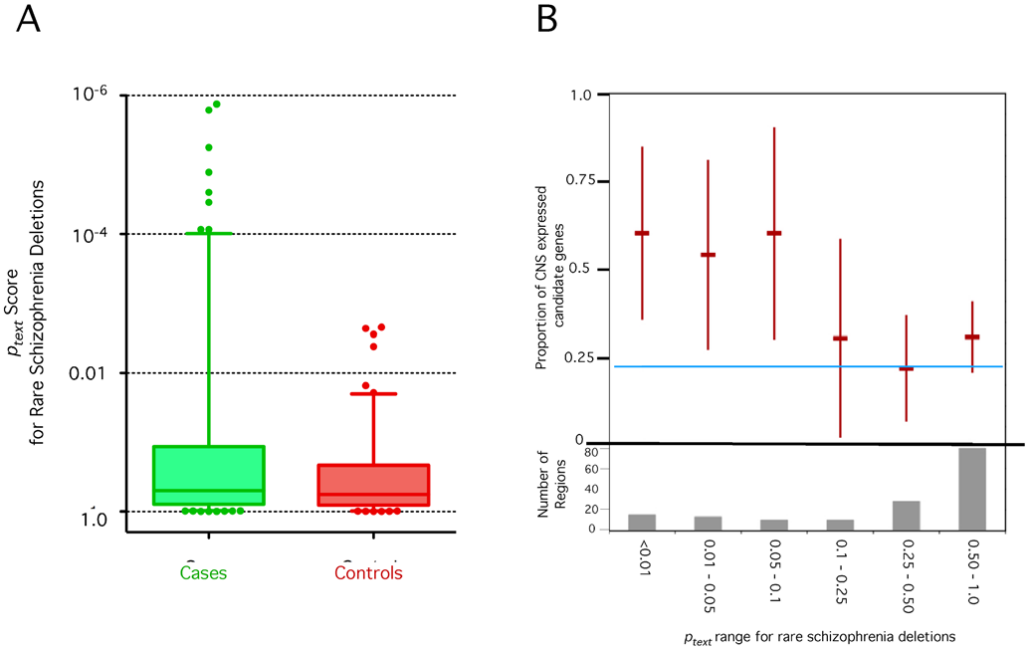
GRAIL identifies a subset of highly connected genes within rare deletions found in Schizophrenia cases. (A) Case deletions versus control deletions. Here we plot the results of the separate GRAIL analyses conducted on the deletions observed in schizophrenia cases and controls. Case deletion *p_text_* scores are displayed in red; control deletion *p_text_* scores are displayed in green. The line in each category in the middle of the box represents the median GRAIL *p_text_* score. The box represents the 25–75% range. The bars represent the 5–95% range. Additional scores outside the range are individual plotted. (B) Text-based GRAIL significance score tracks with CNS specific expression. We partition case-only deletions by their GRAIL scores. For each range of GRAIL *p_text_* scores, we assess the candidate genes selected by GRAIL for CNS expression. The upper portion of this plot illustrates the fraction of those candidate genes that demonstrate preferential CNS expression along with 95% confidence intervals. The blue line represents the total fraction of genes that are preferentially CNS expressed. For the most compelling GRAIL scores, the candidate genes are significantly enriched for CNS expression compared to what would be expected from a random group of genes. The lower portion of the plot is a histogram.

We applied GRAIL separately to both the case and control sets of deletions. In the case deletions, we identified a subset containing highly connected genes (Figure 4A). Specifically, 12 of the 165 regions obtain *p_text_* scores<0.001 with text-similarity (Table 3). The top keywords suggest some common biological underlying functions: ‘phosphatase’, ‘glutamate’, ‘receptor’, ‘cadherin’, and ‘neurons’. In contast, we did not identify any regions with significantly related genes in the corresponding list of deletions; out of a total 124 regions, none obtained *p_text_* scores<0.001 (see Table S8). This represents a significant enrichment within the cases (*p* = 0.01, one Fisher's exact text).

**Table 3:** Rare or de novo schizophrenia case deletions.

CHR	Start	Stop	*p_text_*	Candidate Gene
7	77,788,564	78,591,795	0.0000013	*MAGI2*
11	*83,680,969	83,943,977	0.0000016	*DLG2*
3	65,781,878	65,975,330	0.0000057	*MAGI1*
11	99,153,400	99,286,239	0.000013	*CNTN5*
4	*87,919,851	88,032,640	0.000025	*PTPN13*
18	8,054,730	8,257,748	0.000035	*PTPRM*
3	*7,177,597	7,314,117	0.000087	*GRM7*
3	7,043,889	7,145,741	0.000087	*GRM7*
6	146,418,079	146,525,433	0.00013	*GRM1*
7	125,707,286	126,050,230	0.00015	*GRM8*
9	9,485,226	9,644,834	0.00024	*PTPRD*
7	3,759,288	4,087,229	0.00033	*SDK1*
3	*197,224,662	198,573,215	0.0011	*DLG1*
15	27,015,263	28,173,703	0.0014	*TJP1*
5	31,250,352	32,213,541	0.0033	*PDZD2*
19	10,231,490	10,493,592	0.0048	*ICAM5*
2	112,407,513	112,512,196	0.014	*MERTK*
5	19,570,562	19,843,415	0.018	*CDH18*
6	145,876,484	146,009,981	0.019	*EPM2A*
7	145,321,439	145,461,533	0.019	*CNTNAP2*
5	63,115,468	63,431,545	0.023	*HTR1A*
14	66,287,336	66,470,393	0.025	*GPHN*
18	56,109,430	56,255,536	0.029	*MC4R*
5	106,805,717	107,026,020	0.03	*EFNA5*
2	233,029,864	233,134,571	0.031	*ALPPL2*
20	2,923,491	3,618,945	0.034	*PTPRA*
1	72,287,807	72,439,333	0.037	*NEGR1*
7	94,306,868	94,497,412	0.044	*PPP1R9A*
7	157,378,450	157,569,847	0.046	*PTPRN2*
1	*144,943,150	146,292,286	0.047	*ACP6*

Here we list all of the deletions that GRAIL identified as most related to other deleted genes (*p_text_*<0.05). For each deletion we list the chromosome, the range of the deletion, the GRAIL *p*-value for the region, and the best candidate gene in the region identified by GRAIL. Most genomic coordinates are listed in HG17. *HG18 coordinates.

We then sought independent assessment of the biological relationship of the genes highlighted by GRAIL by examining the extent to which these genes demonstrate preferential expression in CNS tissues using a publicly available tissue atlas [30]. Here we define preferential expression as median CNS tissue expression significantly greater than in other tissues (*p*<0.01 by one-tailed rank-sum test). Considering the entire set, case-deletions are not enriched for genes preferentially expressed in the CNS (22% are preferentially expressed in the CNS, compared to 25% of control-deletion genes). However, considering the subset of genes indentified by GRAIL (*p_text_*<0.01), 60% (9 of 15 genes) are preferentially CNS expressed. Furthermore, the fraction of genes with preferential CNS expression correlates inversely with the significance of the GRAIL score (Figure 4B). Regions that GRAIL assigns non-significant scores to, do not demonstrate any compelling enrichment for CNS expressed genes.

## Discussion

We have presented an automated text-based strategy to take a list of disease regions and identify those regions with significantly inter-related genes. In the process it recognizes the likely candidate gene in each disease region. It makes no assumptions about the phenotype being studied or underlying pathways that might be presumed to be relevant to a disease state. While in principle a diligent investigator could potentially examine the literature related to all potentially associated genes and arrive at the same conclusions, they are unlikely in practice be able to work with the same efficiency and objectivity as the approach outlined here. In the schizophrenia application, for example, we objectively interpret and analyze the relationship between over 500 genes.

We present data that GRAIL can identify common SNPs that subsequently validate in replication genotyping. We have demonstrated superior performance in this application to other methods. This approach could have widespread application to follow-up GWA study results and offers a mechanism to prioritize the hundreds of SNPs that are expected to achieve an intermediately significant level of association (10^−5^<*p*<10^−3^). As far as we are aware – this is the first successful prediction of the outcome of a GWA validation study.

GRAIL offers the greatest value in situations where disease regions are being considered that are difficult to validate, for example rare deletions. The ability to genetically validate any individual rare deletion is challenged given the limited power afforded by the size of available patient collections. In schizophrenia the excess of rare deletions has now been well documented – but it had been difficult to connect these rare deletions to a specific pathway. We identified a subset of related genes that have functions that are plausibly related to schizophrenia. As other diseases emerge where rare variants play a role in the genetic architecture, our approach may provide a crucial first step to put context to genetic findings.

### Connecting seemingly unrelated genes through text

The main strength of GRAIL is its ability to link genes through text that may not yet have an established common pathway or process. Consider the *IRGM* gene association to Crohn's disease – for which GRAIL found strong evidence (uncorrected *p_text_* = 0.0011). GRAIL's text-based similarity metric recognize the significant connections between *IRGM* and four other validated or intermediate region genes: *IRF1*, *IL12B*, *IRF8*, and *SP110*. *IRGM* is not readily connected to these genes in a well-defined pathway and, in-fact, is not referenced together with them in any abstracts; furthermore no *IRGM* interactions are listed in Entrez at all. Yet they all are involved in the host response to *Mycobacterium* and possibly other intracellular infections by macrophages. The top keywords describing the connections between *IRGM* and these genes were ‘macrophages’, ‘tuberculosis’ and ‘mycobacterium’. The *IRGM* gene has been shown experimentally to eliminate intracellular *Mycobacterium tuberculosis* via autophagy [31]. The *IRF1* homolog studies in mouse have demonstrated its role in intra-cellular nitrous oxide production, necessary to fight *Mycobacterium* infections [32]. Individuals with loss of function *IL12B* mutations have been found with increased susceptibility to *Mycobacterium* infections [33] and knock out mice have demonstrated increased susceptibility to infection [34],[35]. A *SP110* mouse homolog has been shown to mediate innate immunity in fighting intra-cellular *Mycobacterium tuberculosis* infection [36]. GRAIL is able to identify this common underlying similarity between these genes, and assign a significant score to *IRGM*, while at the same time revealing what may be an important pathway in Inflammatory Bowel Disease. Other strategies depending on interaction networks or functional databases may struggle to detect these relationships.

### Identifying disease genes within a region

GRAIL systematically identifies a single gene within a disease region as the likely disease gene. We highlight two interesting examples from the height data of previously unrecognized potentially causative genes. The first example is the rs42046 SNP on chromosome 7 region implicating five genes. The genetic studies that identified this region had suggested *CDK6* as the likely causative gene [2]–[4]. However, GRAIL found greatest evidence in support of *PEX1* (uncorrected *p_text_* = 0.0084). When we compare the most compelling of these genes, *PEX1*, to candidates from the other 41 SNPs with our text-based metric, we found it to be most related to a gene in a height-associated SNP on chromosome 8, *PEX2* (*PXMP3*). The protein products of both *PEX1* and *PEX2* are involved in peroxisome biogenesis and are implicated in a genetic disease associated craniofacial and skeletal abnormalities (Zellweger's syndrome) [37]–[39]. While it may be a coincidence that these two closely related genes are associated by chance, it is certainly possible that peroxisome biogenesis represents a previously unrecognized height pathway. The second example is the rs10935120 SNP on chromosome 3, implicating three genes; the genetic study that had identified this gene had suggested *ANAPC13* as the likely candidate in the region [4]. However, GRAIL identified the *KY* gene as the most likely disease gene (*p*_text_ = 0.04). In fact, a mutation in the *KY* gene causes spinal scoliosis in a mouse model [40], and the *KY* protein product interacts with sarcomeric cytoskeletal proteins [41]. While these literature-based hypotheses may be obvious to a few specialized researchers, the strength of GRAIL is that it is able to suggest these connections in a systematic and objective manner from the entirety of the published literature.

### Genes deleted in schizophrenia suggest relevant neuronal processes

We consider closely the subset of related genes identified by GRAIL from rare deletions in patients with schizophrenia. Schizophrenia is a disorder characterized by hallucinations, delusions, cognitive deficits and apathy. The molecular basis of the symptom complex associated with the disorder is largely unknown. However accumulating evidence suggest that dysregulation of synaptic activity and abnormalities in neuronal development and migration may contribute to the pathophysiology of schizophrenia [42]. Many of the highest scoring genes recovered by GRAIL within the deleted regions in cases (Table 3) are localized to the postsynaptic membrane/signaling complex that propagate signals resulting in changes synapse function and downstream gene expression/transcription. The *DLG2* gene product interacts at postsynaptic sites to form a multi-meric scaffold for the clustering of receptors, ion channels, and associated signaling proteins. *MAGI1* and *MAGI2* both encode post-synaptic scaffolding molecules involved in cell adhesion and signaling [43],[44]. Furthermore, glutamatergic neurotransmission is implicated through the selection of *GRM1*, *GRM7*, and *GRM8*. Many of the most significant candidate genes identified by GRAIL are involved in neuronal development, cell-cell adhesion and axon guidance. *CNTN5* is an immunoglobulin super-family membrane-anchored neuronal protein that is also an adhesion molecule [45]. It may play a role in the developing nervous system [46]. The *SDK1* gene expresses a synaptic adhesion protein [47] that guides axonal terminals to specific synapses in developing neurons. The *PTPRM* encodes a neuronally expressed protein tyrosine phosphatase that mediates cell-cell aggregation and is involved in cell-cell adhesion [48],[49].

### Competing methods

The most critical technical difference between GRAIL and other strategies is that it does not use any strict definitions of gene functions or interactions, but rather uses a metric of relatedness that allows for a relatively broad range of freedom with which to connect genes. While GRAIL will certainly identify relationships between genes known to be in a common pathway, it goes beyond that, and can allow less strict evidence. In fact, it is even able to identify relatedness between genes that have no established common pathways or article co-citations! In contrast, other strategies start with static gene relationships—such as (1) pre-constructed molecular networks [12],[16] or sets of gene with common function [11],[15] or (2) a subset of functions identified as relevant to disease either by the user [17] or by mining the published text [14]. In a head to head match up against four other methods that we were able to obtain implemented versions of, GRAIL demonstrated superior performance in predicting Crohn's associated SNPs (see Text S1, Figure S2, Table S5, and Table S6).

### Limitations to assessing gene relatedness with text

While we have shown the promise of text-based similarity in identifying regions and the genes within them that are part of a larger biological pathway, we note that this strategy's effectiveness is wholly contingent on the completeness of the scientific text. It could be biased towards subsets of genes and pathways that are particularly well studied, and against poorly studied ones. In many of the cases that we illustrate, there are regions that could not be connected – for example, GRAIL fails to connect 5 validated Crohn's SNPs that obtain *p_text_* scores>0.5 (Figure 3B). These regions might have been missed since the relevant gene is either poorly studied, or even if the gene is well studied, the relevant function of that gene is not well documented in the text. An alternative possibility is that the SNP is tagging non-genic regulatory elements. Additionally, the SNP may be the first discovered representative association for a critical pathway, not represented by other SNP associations – and therefore cannot be connected to them. In this case future discoveries will clarify the significance of that association.

In cases where there is no apparent published connection between associated genes, other similarity metrics based on experimentally derived data, such as gene expression, protein-protein interactions and transcription factor binding sites could also complement the text-based approaches presented here. In fact, we demonstrate how annotation-based metrics or gene expression-based metrics are able to identify a subset of the associated SNPs in lipid metabolism. As these and other metrics are optimized, they could be used in conjunction with the novel GRAIL statistical framework that we present here to help understand gene relationships.

## Methods

### Scoring regions for functional relatedness

The Gene Relationships Among Implicated Loci (GRAIL) has four basic steps that are outlined below. It has two input sets of disease regions: (1) a collection of *N_SEED_ seed* regions (SNPs or CNVs) and (2) a collection of *N_QUERY_ query* regions. Genes in *query* regions are evaluated for relationships to genes in *seed* regions, and *query* regions are then assigned a significance score. In most applications we are examining a set of regions for relationships between implicated genes, the *query* regions and the *seed* regions are identical. In other circumstances where we have a set of putative regions that are being tested against validated ones, the putative regions are defined as *query* regions, and the validated ones are defined as *seed* regions.

#### Step 1. Defining disease regions and identifying overlapping genes

For each *query* and *seed* SNP we find the furthest neighboring SNPs in the 3′ and 5′ direction in LD (r^2^>0.5, CEU HapMap [50]). We then proceed outwards in each direction to the nearest recombination hotspot [51]. The interval between those two hotspots, which would include the SNP of interest and all SNPs in LD, is defined as the disease region. The associated SNP could feasibly be tagging a stronger SNP signal from another SNP in that region. All genes that overlap that interval are considered implicated by the SNP. If there are no genes in that region, the interval is extended an additional 250 kb in either direction; we chose 250 kb as that distance since that is a range in which non-coding variants might express gene regulation [52]. For each *query* and *seed* CNV we define an interval that represents the deleted or duplicated region—all genes that overlap that interval are associated with the CNV for testing.

#### Step 2. Ranking gene relatedness

For each gene near a *query* region, we rank all human genes for relatedness. Ranking may be based on text similarity, or other metrics (see below for examples). Rank values range from 1 (most related) to *N_G_* (least related), where *N_G_* is the number of available human genes, in our application is 18,875 (see Table 1).

#### Step 3. Scoring candidate genes against regions

To avoid double counting nearby regions, we first combine any *seed* regions sharing one or more genes. For a given gene *g* in a *query* region, we examine the degree of similarity to any of the *n_s_* genes in a given *seed* region *s*. To ensure independence, we only look at a *seed* region *s*, if it does not share a single gene with the *query* region that gene *g* is contained in.

We identify in each region *s*, the rank of the most similar (or lowest ranking) gene in it to gene *g*, *R_g,s_*. We convert the rank to a proportion:
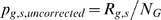
To transform this proportion to a uniformly distributed entity under the null, we recognize that *R_g,s_* was the lowest rank selected from *n_s_* genes – and we correct accordingly for multiple hypothesis testing:
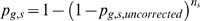
Now we identify those seed regions where *p_g,s_* is less than a pre-specified threshold *p_f_* as regions connected to gene *g*. For all applications presented here *p_f_* is arbitrarily set to 0.1. The number of *seed* regions containing at least one gene exceeding this threshold, *n_hit_*, can be approximated under a random model with a Poisson distribution.

We assign a greater weight to those cases where there is greater similarity; that is in the cases where *p_g,s_* is particularly small:
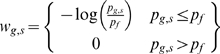
Under a random model, if *p_g,s_*<*p_f_* , *p_g,s_* should range approximately uniformly from 0 to *p_f_*. Therefore, under these circumstances *w_g,s_* can be modeled approximately with a gamma distribution.

For each candidate gene, *g*, we tally the number of *seed* regions that contain a highly related gene into a weighted count, *c_g_*:
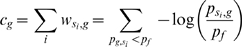



After testing gene *g* across *N_SEED_ seed* regions for related genes, the probability of a score exceeding *c_g_* under the null, *p_g_*, can be approximated:
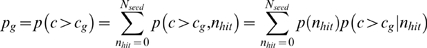
Where *n_hit_* is the number of seed regions connected to gene *g*. Since under the null model the probability of a connected region by chance is always *p_f_*, we can estimate its probability distribution of *n_hit_* with a Poisson distribution:
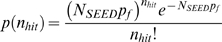
Since, *c_g_*, is the sum of the log of *n_hit_* independent uniformly distributed values ranging from 0 to 1, for a fixed value of *n_hit_* we can calculate the distribution of *c_g_* with a cumulative gamma distribution:
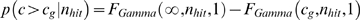
Since *n_hit_* is always an integer, the *F_Gamma_* term can be simplified:
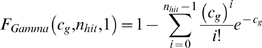
Therefore, we can be further simplified:
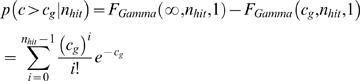
Putting this together:
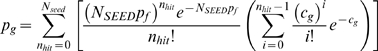



#### Step 4. Scoring regions

Finally, for each *query* region we identify the best scoring gene within it. A significance score for the *query* region, *p_q_*, is based on the *p*-value of that gene, *p_g_*, corrected for multiple hypothesis testing. Assuming the region has *n_q_* genes within it:
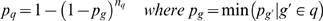



### Assessing gene relatedness with text-based similarity

We measure relatedness between genes using similarity in published text from gene references. We first obtain article abstracts from Pubmed. We downloaded all abstracts on December 16, 2006. For each gene, we identified and downloaded abstract references listed in Entrez Gene [23]; additionally, we downloaded Entrez Gene abstract references for gene orthologs listed in Homologene [53]. We removed those articles referencing more than 10,000 genes. Only the title (TI) and abstract (AB) fields were included for further text processing. We defined a vocabulary consisting of only those terms appearing in 40 or more abstracts, and fewer than 130,000; this resulted in a vocabulary of 23,594 terms. For each abstract *j* we create a vector of term frequencies, *tf_ij_*, representing the number of times each term *i* appears within it. Term frequencies are transformed into weights, *w_ij_*, according to a standard inverse document frequency scheme [54]:
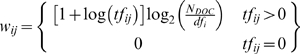
where *N_DOC_* is the total number of documents, and *df_i_* (or document frequency) is the number of documents the term *i* appears in. This scheme emphasizes rare words, and de-emphasizes more common words.

For every gene, we define an averaged term-vector, which is an average of weighted term vectors from gene references and homologous gene references. Abstracts are weighted according to the number of genes they reference; articles referencing many genes are down-weighted to mitigate their influence:
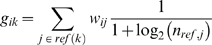
where *g_ik_* is a the weighted count of term *i* for gene *k*, *j* is a document reference for gene *k*, and document *j* references *n_ref,j_* genes. For a given gene *i* these *g_ik_* terms define a gene-text vector. The gene text vectors are normalized, so that their euclidean length is 1. Pairwise gene relatedness can be calculated as the dot product between two normalized term vectors for genes.

### Keywords

To assign keywords to a collection of query regions, we first identify the single candidate genes with the best GRAIL *p*_text_ from each region. We then eliminate those regions where the uncorrected GRAIL score for the gene is *p*_text_>0.2. We restrict keywords to those that appear in >500 documents, contain >3 letters, and have no numbers. For each term, *i*, we calculate a score which is the difference between averaged term frequencies among candidate genes and all genes:
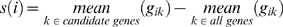
The top twenty highest scoring terms are selected as keywords.

### Annotation based relatedness

We defined a relatedness metric between genes based on similarity in Gene Ontology annotation terms [27]. We downloaded Gene Ontology structure and annotations on December 19, 2006. In addition to human gene GO annotations, we added orthologous gene annotations. Since GO is a hierarchically structured vocabulary, for each gene annotation we also added all of the more general ancestral terms. This resulted in a total of 843,898 annotations for 18,050 genes with 10,803 unique GO terms; this corresponds to a median of 40 terms per gene. We weighted annotations proportionally to the inverse of their frequency, so common annotations received less emphasis. We used a weighting scheme analogous to the one we used for word weighting:
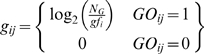
where *g_ij_* represented the weighted code *i* for gene *j*, *N_G_* is the total number of genes, and *gf_i_* (or GO frequency) is the number of genes annotated with the term *i*. Gene relatedness was the correlation between these weighted annotation vectors.

### Gene expression based relatedness

To calculate gene relatedness based on expression we downloaded the Novartis Gene Expression Atlas [28]. The data set consists of measurements for 33,689 probes across 158 conditions. Probes were averaged into 17,581 gene profiles. Gene relatedness was calculated as the correlation between expression vectors.

### Lipid and height applications

We applied GRAIL to score 19 lipid-associated SNPs and separately to score 42 height-associated SNPs. Specific SNPs are listed in Table S1 and Table S2. We used the SNP sets as both the *seed* and the *query* set to look for relatedness between genes across regions. We scored SNPs separately using text, annotation, and expression similarity metrics. We compiled the best candidate genes and scores for the SNP regions.

### Crohn's disease application

Prior to replication, we had access to 74 independent SNP regions that had emerged from a meta-analysis of Crohn's Disease. All 74 SNPs were used as both the *query* set and as the *seed* set into GRAIL. We assessed whether those SNPs that replicated had different text-based significance values than those that fail to replicate. To identify additional regions of interest, we identified the next 75 most significant regions in the Crohn's disease meta-analysis – they were used in GRAIL as a *query* set; for the *seed* set included all SNPs that did not fail in replication.

### Schizophrenia application

We identified singleton deletions or confirmed de novo deletions reported by one of three groups. We selected those deletions that were in cases only or in controls only, were at least 100 kb large, and included at least one gene. We obtained singleton deletions online published by the International Schizophrenia Consortium (2008) at [8]. We obtained de novo deletions published by Xu et al (2008) from Table 1 [10]. We obtained singleton deletions published in Walsh et al (2008) from Table 2 [9]. We identified a total of 165 case-only deletions and 122 control-only deletions. We applied the GRAIL algorithm separately to case and controls. We speculated that the case deletions might hit genes from a common pathway and GRAIL *p*-values may therefore be enriched for significant scores. On the other hand, we hypothesized that control deletions might be located effectively at random, and so no particular pathway or common function should necessarily be enriched in this collection.

To examine genes for tissue specific expression in the CNS system, we obtained a large publicly available human tissue expression microarray panel (GEO accession: GSE7307) [30]. We analyzed the data using the robust multi-array (RMA) method for background correction, normalization and polishing [55]. We filtered the data excluding probes with either 100% ‘absent’ calls (MAS5.0 algorithm) across tissues, expression values <20 in all samples, or an expression range <100 across all tissues. To represent each gene, we selected the corresponding probe with the greatest intensity across all samples. The data contained expression profiles for 19,088 genes. We included expression profiles from some 96 normal tissues and excluded disease tissues and treated cell lines. We averaged expression values from replicated tissues averaged into a single value. To assess whether genes had differential expression for CNS tissues, we compared the 27 tissue profiles that represented brain or spinal cord to the remaining 69 tissue profiles with a one-tailed Mann-Whitney rank-sum test. Genes obtaining *p*<0.01 were identified as preferentially expressed.

### Evaluation against other published methods

We compared GRAIL's performance in its ability to prospectively predict Crohn's associations to five other published methods. The selection of these methods, and the evaluation is detailed in Text S1.

### Software

An online version of this method is available (http://www.broad.mit.edu/mpg/grail/).

## Supporting Information

Figure S1 GRAIL p-value scores for random SNPs. We scored 100 random groups of 50 SNPs with GRAIL. The y-axis is the fraction of SNPs in the group with values below the threshold, the x-axis lists the specific threshold. For each threshold, we plot the distribution of the fraction of the 50 SNPs below that threshold as a box plot. The bar is the median - the mean value is explicitly listed below the box-plot. The box at each threshold lists the 25%–75% range. The error-bars line depicts the 1.5 inter-quartile range. The black dots illustrate outliers outside the 1.5 inter-quartile range.
(0.39 MB PDF)

Figure S2 Sensitivity versus specificity for prioritization algorithms. We used 5 algorithms to score the 74 most promising putative SNP associations from the Crohn's meta-analysis study. We assessed each algorithm's ability to predict those SNP associations that ultimately validated in follow-up genotyping. For each algorithm, we created a received-operator curve (ROC).
(0.40 MB PDF)

Table S1 19 Lipid regions scored with Text based GRAIL strategy. Here we scored 19 SNPs, associated with lipid metabolism. In the first three columns we list information about the SNP. In the fourth column we list the number of genes in the SNP associated regions. In the fifth column we list the highest scoring gene in the associated region based on GRAIL using a text-based metric. In the sixth column we list the *p_text_* values for the associated regions. We have bolded those candidate genes that are known likely causative gene. The seventh and eight columns list similar results for GRAIL with an GO annotation-based metric. The ninth and tenth columns list similar results for GRAIL with an expression-based metric.
(0.15 MB DOC)

Table S2 42 Height regions scored with Text based GRAIL strategy. Here we scored 42 SNPs, associated with height. In the first three columns we list information of the SNP. In the fourth column we list the number of genes in the SNP associated regions. In the fifth column we list the highest scoring gene in the associated region for the SNP based on GRAIL using a text-based metric. In the sixth column we list the *p_text_* values for the associated regions. The seventh and eight columns list similar results for GRAIL with an annotation-based metric. The ninth and tenth columns list similar results for GRAIL with an expression-based metric.
(0.28 MB DOC)

Table S3 Keywords for Lipid and Height SNPs. We identified keywords associated with lipid and height associated SNPs; here we list the top 20.
(0.06 MB DOC)

Table S4 Crohn's Disease SNPs from a meta-analysis of GWA studies. Here we list GRAIL results and summarize genotyping results for Crohn's disease SNPs. These 74 SNPs emerged from a meta-analysis and as a result of replication genotyping, they were either validated (A), indeterminate (B), or failed (C). For each of the regions we list the SNP ID and the chromosome in the second and third column. In the fourth column we list the final combined association significance score of the SNP to the Crohn's disease. In the fifth, sixth, and seventh columns we list GRAIL results including the number of genes in the region, the best candidate gene, and the text-based significance score for the region.
(0.21 MB DOC)

Table S5 Algorithms to prioritize candidate genes. Our search of the literature identified nine algorithms that could be used to prioritize genes for replication. Four methods require no user-specified disease information (supervised), and five require some disease information from the user. We list in each row the name of the disease, the website, the necessary genetic data, the functional data used to prioritize genes, the disease-specific information that must be included, and the availability of the method.
(0.09 MB DOC)

Table S6 Performance measures for prioritization algorithms. We used five algorithms (column 1) to score putatively associated SNPs from the Crohn's meta-analysis. After calculating an ROC curve for each algorithm, we calculated the AUC (column 2). We also calculated a p-value with a one-tailed rank-sum test comparing the median rank of the validated SNPs to the median rank of the failed SNPs (column 2).
(0.04 MB DOC)

Table S7 Other promising regions in Crohn's Disease GWA meta-analysis. Information about the top six regions identified by GRAIL from the next 75 most significant regions from the Crohn's GWA study. All associations are indeterminate, and association p-values are taken from the GWA meta-analysis - these regions have not yet been replicated.
(0.05 MB DOC)

Table S8 Rare or de novo schizophrenia control deletions. Here we list all of the deletions that GRAIL identified as most related to other deleted genes (*p_text_*<0.05). For each deletion we list the chromosome, the range of the deletion, the GRAIL p-value for the region, and the best candidate gene in the region identified by GRAIL. Most genomic coordinates are listed in HG17. * HG18 coordinates.
(0.06 MB DOC)

Text S1 A. Random SNP groups; B. Comparison of GRAIL to other related algorithms.
(0.09 MB DOC)
