# Supplementary material for: Identifying Relationships among Genomic Disease Regions: Predicting Genes at Pathogenic SNP Associations and Rare Deletions
Source: PLoS Genet. 2009 Jun 26;5(6):e1000534. doi: 10.1371/journal.pgen.1000534 (PMC2694358; doi:10.1371/journal.pgen.1000534)
Supplement: Figure S1 — GRAIL p-value scores for random SNPs. We scored 100 random groups of 50 SNPs with GRAIL. The y-axis is the fraction of SNPs in the group with values below the threshold, the x-axis lists the specific threshold. For each threshold, we plot the distribution of the fraction of the 50 SNPs below that threshold as a box plot. The bar is the median - the mean value is explicitly listed below the box-plot. The box at each threshold lists the 25%–75% range. The error-bars line depicts the 1.5 inter-quartile range. The black dots illustrate outliers outside the 1.5 inter-quartile range. (0.39 MB PDF) [file pgen.1000534.s001.pdf]

Figure S1

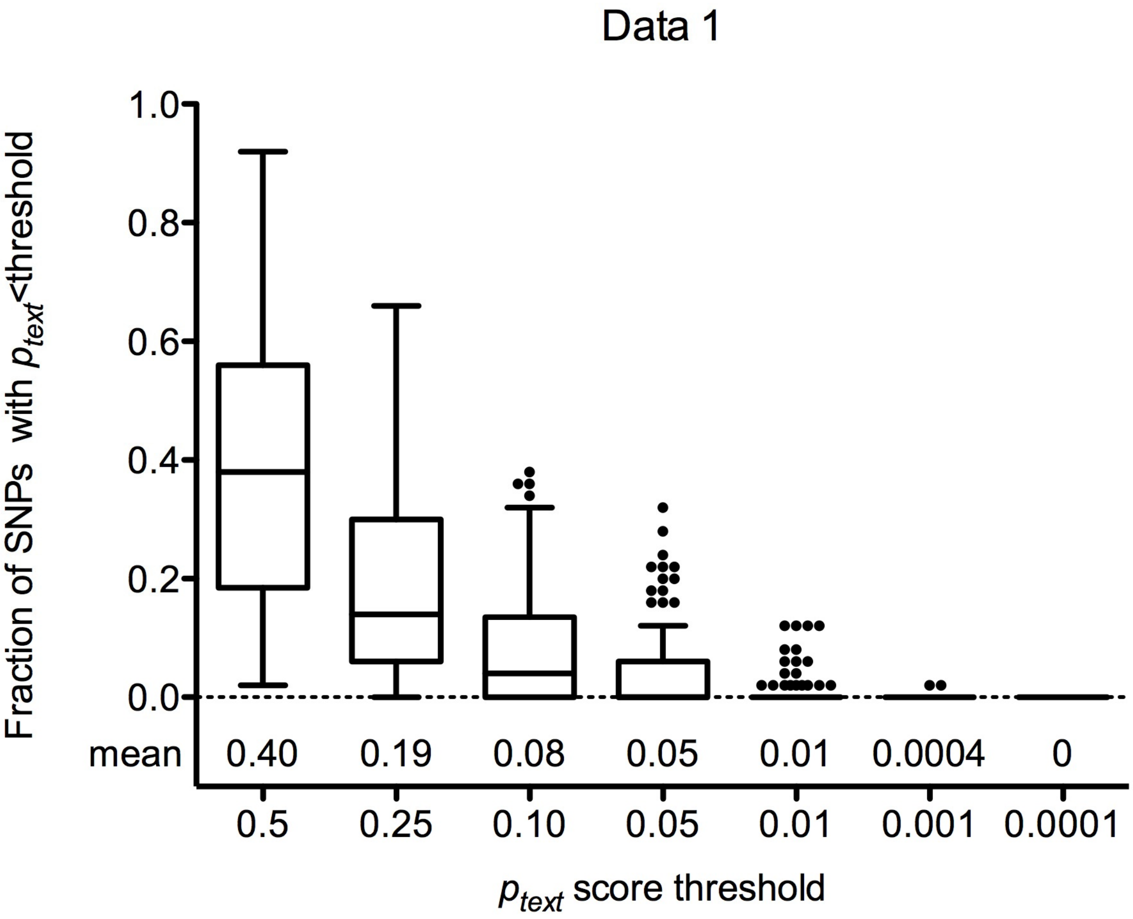

Figure S1. GRAIL p-value scores for random SNPs. We scored 100 random groups of 50 SNPs with GRAIL. The *y*-axis is the fraction of SNPs in the group with values below the threshold, the *x*-axis lists the specific threshold. For each threshold, we plot the distribution of the fraction of the 50 SNPs below that threshold as a box plot. The bar is the median – the mean value is explicitly listed below the box-plot. The box at each threshold lists the 25-75% range. The error-bars line depicts the 1.5 inter-quartile range. The black dots illustrate outliers outside the 1.5 inter-quartile range.
