# Supplementary material for: Identifying Relationships among Genomic Disease Regions: Predicting Genes at Pathogenic SNP Associations and Rare Deletions
Source: PLoS Genet. 2009 Jun 26;5(6):e1000534. doi: 10.1371/journal.pgen.1000534 (PMC2694358; doi:10.1371/journal.pgen.1000534)
Supplement: Figure S2 — Sensitivity versus specificity for prioritization algorithms. We used 5 algorithms to score the 74 most promising putative SNP associations from the Crohn's meta-analysis study. We assessed each algorithm's ability to predict those SNP associations that ultimately validated in follow-up genotyping. For each algorithm, we created a received-operator curve (ROC). (0.40 MB PDF) [file pgen.1000534.s002.pdf]

**FIGURE S2**

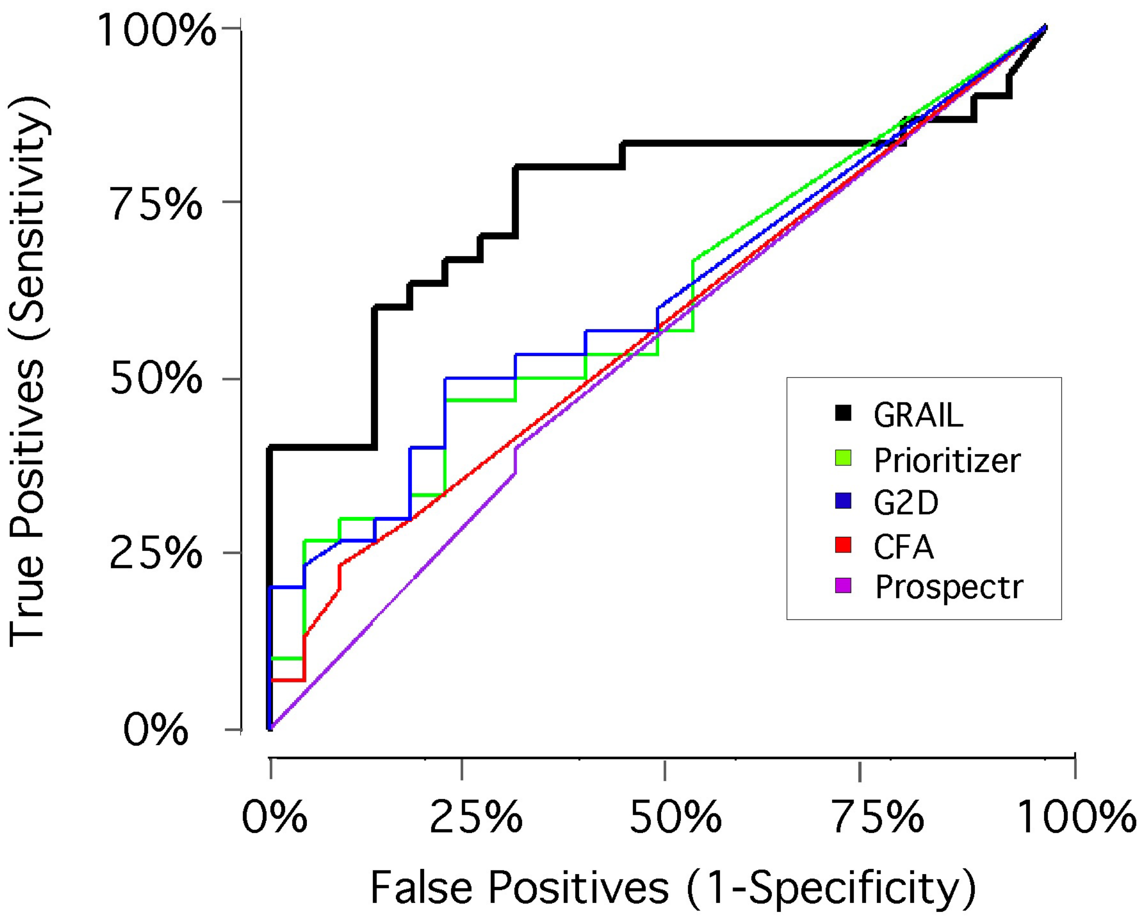


**Figure S2. Sensitivity versus specificity for prioritization algorithms.** We used 5 algorithms to score the 74 most promising putative SNP associations from the Crohn's meta-analysis study. We assessed each algorithm's ability to predict those SNP associations that ultimately validated in follow-up genotyping. For each algorithm, we created a received-operator curve (ROC).
