# Supplementary material for: Identifying Relationships among Genomic Disease Regions: Predicting Genes at Pathogenic SNP Associations and Rare Deletions
Source: PLoS Genet. 2009 Jun 26;5(6):e1000534. doi: 10.1371/journal.pgen.1000534 (PMC2694358; doi:10.1371/journal.pgen.1000534)
Supplement: Table S2 — 42 Height regions scored with Text based GRAIL strategy. Here we scored 42 SNPs, associated with height. In the first three columns we list information of the SNP. In the fourth column we list the number of genes in the SNP associated regions. In the fifth column we list the highest scoring gene in the associated region for the SNP based on GRAIL using a text-based metric. In the sixth column we list the ptext values for the associated regions. The seventh and eight columns list similar results for GRAIL with an annotation-based metric. The ninth and tenth columns list similar results for GRAIL with an expression-based metric. (0.28 MB DOC) [file pgen.1000534.s004.doc]

**Table S**2

| Region | SNP | Chr | N | Text | | Annotation | | Expression | |
| --- | --- | --- | --- | --- | --- | --- | --- | --- | --- |
|  |  |  | (genes) | Implicated Gene | *p_text_* | Implicated Gene | *p_annotation_* | Implicated Gene | *p_expression_* |
| 1 | rs6724465 | 2 | 8 | *IHH* | 5.5E-06 | *IHH* | 0.037 | *NHEJ1* | 0.71 |
| 2 | rs6854783 | 4 | 4 | *HHIP* | 2.6E-05 | *HHIP* | 0.35 | *HHIP* | 0.010 |
| 3 | rs6060373 | 20 | 14 | *GDF5* | 3.2E-05 | *GDF5* | 0.69 | *ERGIC3* | 0.99 |
| 4 | rs1042725 | 12 | 1 | *HMGA2* | 5.1E-05 | *HMGA2* | 0.014 | *HMGA2* | 0.86 |
| 5 | rs10512248 | 9 | 1 | *PTCH1* | 1.2E-04 | *PTCH1* | 6.1E-04 | *PTCH1* | 0.53 |
| 6 | rs12198986 | 6 | 1 | *BMP6* | 1.6E-04 | *BMP6* | 0.11 | *BMP6* | 0.19 |
| 7 | rs967417 | 20 | 1 | *BMP2* | 5.1E-04 | *BMP2* | 0.0044 | *BMP2* | 0.11 |
| 8 | rs3791675 | 2 | 1 | *EFEMP1* | 0.0014 | *EFEMP1* | 0.21 | *EFEMP1* | 0.040 |
| 9 | rs9650315 | 8 | 2 | *PLAG1* | 0.0017 | *PLAG1* | 0.31 | *CHCHD7* | 0.59 |
| 10 | rs8007661 | 14 | 3 | *FBLN5* | 0.0017 | *FBLN5* | 0.30 | *ATXN3* | 0.58 |
| 11 | rs757608 | 17 | 2 | *TBX2* | 0.0020 | *TBX2* | 0.14 | *TBX2* | 0.43 |
| 12 | rs8041863 | 15 | 2 | *ACAN* | 0.0041 | *ACAN* | 0.084 | *ACAN* | 0.39 |
| 13 | rs2814993 | 6 | 11 | *SCUBE3* | 0.0062 | *SNRPC* | 0.44 | *C6orf106* | 0.80 |
| 14 | rs4713858 | 6 | 4 | *FANCE* | 0.016 | *FANCE* | 0.45 | *TEAD3* | 0.26 |
| 15 | rs12986413 | 19 | 6 | *AMH* | 0.022 | *SF3A2* | 0.72 | *C19orf36* | 0.65 |
| 16 | rs11107116 | 12 | 1 | *SOCS2* | 0.024 | *SOCS2* | 0.73 | *SOCS2* | 0.059 |
| 17 | rs2844479 | 6 | 50 | *TNXB* | 0.025 | *CSNK2B* | 0.25 | *C6orf47* | 1.0 |
| 18 | rs4800148 | 18 | 1 | *CABLES1* | 0.036 | *CABLES1* | 0.22 | *CABLES1* | 0.52 |
| 19 | rs1776877 | 6 | 2 | *HMGA1* | 0.038 | *HMGA1* | 0.040 | *C6orf1* | 0.65 |
| 20 | rs42046 | 7 | 5 | *PEX1* | 0.041 | *CDK6* | 0.77 | *DKFZP564O0523* | 0.91 |
| 21 | rs10935120 | 3 | 3 | *KY* | 0.11 | *ANAPC13* | 0.40 | *CEP63* | 0.95 |
| 22 | rs1390401 | 1 | 4 | *MPN2* | 0.12 | *JMJD4* | 0.098 | *JMJD4* | 0.88 |
| 23 | rs4549631 | 6 | 1 | *C6orf173* | 0.14 | *C6orf173* | 0.20 | N/A | N/A |
| 24 | rs7846385 | 8 | 2 | *PXMP3* | 0.15 | *ZFHX4* | 0.53 | *ZFHX4* | 0.87 |
| 25 | rs4533267 | 15 | 1 | *ADAMTS17* | 0.15 | *ADAMTS17* | 0.0089 | *ADAMTS17* | 0.61 |
| 26 | rs798544 | 7 | 2 | *AMZ1* | 0.19 | *AMZ1* | 0.10 | *AMZ1* | 0.64 |
| 27 | rs6686842 | 1 | 3 | *SCMH1* | 0.22 | *CTPS* | 0.67 | *SLFNL1* | 0.53 |
| 28 | rs12735613 | 1 | 1 | *SPAG17* | 0.27 | *SPAG17* | 0.035 | *SPAG17* | 0.45 |
| 29 | rs10946808 | 6 | 16 | *HIST1H3D* | 0.286 | *HIST1H2BE* | 0.29 | *HIST1H2BD* | 0.19 |
| 30 | rs4794665 | 17 | 1 | *DGKE* | 0.30 | *DGKE* | 1.0 | *DGKE* | 0.52 |
| 31 | rs3760318 | 17 | 6 | *CENTA2* | 0.35 | *C17orf42* | 0.22 | *ATAD5* | 0.82 |
| 32 | rs314277 | 6 | 1 | *LIN28B* | 0.380 | *LIN28B* | 0.44 | *LIN28B* | 0.48 |
| 33 | rs4896582 | 6 | 1 | *GPR126* | 0.410 | *GPR126* | 1.0 | *GPR126* | 0.17 |
| 34 | rs678962 | 1 | 1 | *DNM3* | 0.419 | *DNM3* | 0.96 | *DNM3* | 0.42 |
| 35 | rs11205277 | 1 | 15 | *HIST2H3A* | 0.466 | *BOLA1* | 0.11 | *MTMR11* | 0.74 |
| 36 | rs3116602 | 13 | 1 | *DLEU7* | 0.50 | N/A | N/A | N/A | N/A |
| 37 | rs6440003 | 3 | 2 | *ZBTB38* | 0.73 | *ZBTB38* | 0.47 | *ZBTB38* | 0.61 |
| 38 | rs10906982 | 15 | 1 | *ADAMTSL3* | 0.76 | *ADAMTSL3* | 0.0044 | *ADAMTSL3* | 0.84 |
| 39 | rs8099594 | 18 | 3 | *DYM* | 0.815 | *RPL17* | 0.27 | *DYM* | 0.13 |
| 40 | rs4743034 | 9 | 1 | *ZNF462* | 0.86 | *ZNF462* | 0.64 | *ZNF462* | 0.21 |
| 41 | rs16896068 | 4 | 3 | *NCAPG* | 0.98 | *LCORL* | 0.10 | *NCAPG* | 0.73 |
| 42 | rs2274432 | 1 | 2 | *C1orf19* | 0.997 | *C1orf19* | 0.63 | *C1orf19* | 0.55 |

**Table S2. 42 Height regions scored with Text based GRAIL strategy.** Here we scored 42 SNPs, associated with height. In the first three columns we list information of the SNP. In the fourth column we list the number of genes in the SNP associated regions. In the fifth column we list the highest scoring gene in the associated region for the SNP based on GRAIL using a text-based metric. In the sixth column we list the *p_text_* values for the associated regions. The seventh and eight columns list similar results for GRAIL with an annotation-based metric. The ninth and tenth columns list similar results for GRAIL with an expression-based metric.
