# Supplementary material for: Identifying Relationships among Genomic Disease Regions: Predicting Genes at Pathogenic SNP Associations and Rare Deletions
Source: PLoS Genet. 2009 Jun 26;5(6):e1000534. doi: 10.1371/journal.pgen.1000534 (PMC2694358; doi:10.1371/journal.pgen.1000534)
Supplement: Table S3 — Keywords for Lipid and Height SNPs. We identified keywords associated with lipid and height associated SNPs; here we list the top 20. (0.06 MB DOC) [file pgen.1000534.s005.doc]

**Table S3**

| Rank | **SERUM LIPID** | **HEIGHT** |
| --- | --- | --- |
| 1 | lipoprotein | hedgehog |
| 2 | cholesterol | histone |
| 3 | lipase | bone |
| 4 | apolipoprotein | cartilage |
| 5 | triglyceride | growth |
| 6 | plasma | morphogenetic |
| 7 | hepatic | peroxisomal |
| 8 | apoe | peroxisome |
| 9 | density | group |
| 10 | lipid | matrix |
| 11 | lipoproteins | development |
| 12 | mice | mice |
| 13 | atherosclerosis | differentiation |
| 14 | levels | retinal |
| 15 | metabolism | signaling |
| 16 | liver | differentiation |
| 17 | glucose | complementation |
| 18 | reductase | expression |
| 19 | subjects | matrix |
| 20 | triglycerides | proteases |

**Table S3. Keywords for Lipid and Height SNPs**. We identified keywords associated with lipid and height associated SNPs; here we list the top 20.
