# Supplementary material for: Identifying Relationships among Genomic Disease Regions: Predicting Genes at Pathogenic SNP Associations and Rare Deletions
Source: PLoS Genet. 2009 Jun 26;5(6):e1000534. doi: 10.1371/journal.pgen.1000534 (PMC2694358; doi:10.1371/journal.pgen.1000534)
Supplement: Table S4 — Crohn's Disease SNPs from a meta-analysis of GWA studies. Here we list GRAIL results and summarize genotyping results for Crohn's disease SNPs. These 74 SNPs emerged from a meta-analysis and as a result of replication genotyping, they were either validated (A), indeterminate (B), or failed (C). For each of the regions we list the SNP ID and the chromosome in the second and third column. In the fourth column we list the final combined association significance score of the SNP to the Crohn's disease. In the fifth, sixth, and seventh columns we list GRAIL results including the number of genes in the region, the best candidate gene, and the text-based significance score for the region. (0.21 MB DOC) [file pgen.1000534.s006.doc]

**Table S4A**

| Region | SNP | Chr | *p_association_* | N (genes) | Implicated Gene | Text-Based *p*-value | Replication Study Result |
| --- | --- | --- | --- | --- | --- | --- | --- |
| 1 | rs11465804 | 1 | 3.3E-63 | 1 | *L23R* | 0.00094 | VALIDATED |
| 2 | rs3828309 | 2 | 1.2E-32 | 4 | *USP40* | 0.019 | VALIDATED |
| 3 | rs17234657 | 5 | 3.4E-27 | 1 | *PTGER4* | 0.29 | VALIDATED |
| 4 | rs2066845 | 16 | 1.5E-24 | 3 | *NOD2* | 0.00010 | VALIDATED |
| 5 | rs10995271 | 10 | 2.2E-20 | 1 | *ZNF365* | 0.92 | VALIDATED |
| 6 | rs2188962 | 5 | 1.2E-18 | 9 | *IRF1* | 0.0026 | VALIDATED |
| 7 | rs2542151 | 18 | 2.6E-17 | 1 | *PTPN2* | 0.47 | VALIDATED |
| 8 | rs11190140 | 10 | 1.5E-16 | 2 | *NKX2-3* | 0.33 | VALIDATED |
| 9 | rs11747270 | 5 | 1.7E-16 | 3 | *IRGM* | 0.0032 | VALIDATED |
| 10 | rs3764147 | 13 | 1.0E-13 | 2 | *C13orf31* | 1.00 | VALIDATED |
| 11 | rs10045431 | 5 | 1.9E-13 | 1 | *IL12B* | 0.00066 | VALIDATED |
| 12 | rs2301436 | 6 | 5.2E-13 | 3 | *CCR6* | 0.0052 | VALIDATED |
| 13 | rs3197999 | 3 | 5.8E-13 | 32 | *ARIH2* | 0.46 | VALIDATED |
| 14 | rs744166 | 17 | 3.4E-12 | 2 | *STAT3* | 0.023 | VALIDATED |
| 15 | rs11584383 | 1 | 7.2E-12 | 3 | *KIF21B* | 1.00 | VALIDATED |
| 16 | rs7746082 | 6 | 1.2E-10 | 2 | *PRDM1* | 0.60 | VALIDATED |
| 17 | rs4263839 | 9 | 1.3E-10 | 2 | *TNFSF8* | 0.008 | VALIDATED |
| 18 | rs11175593 | 12 | 1.5E-10 | 2 | *LRRK2* | 0.27 | VALIDATED |
| 19 | rs6908425 | 6 | 4.5E-10 | 1 | *CDKAL1* | 0.42 | VALIDATED |
| 20 | rs7927894 | 11 | 6.6E-10 | 1 | *C11orf30* | 0.28 | VALIDATED |
| 21 | rs762421 | 21 | 7.0E-10 | 1 | *ICOSLG* | 0.0023 | VALIDATED |
| 22 | rs2274910 | 1 | 7.3E-10 | 2 | *ITLN2* | 1.00 | VALIDATED |
| 23 | rs9286879 | 1 | 7.7E-10 | 4 | *TNFSF18* | 0.0042 | VALIDATED |
| 24 | rs17582416 | 10 | 8.9E-10 | 4 | *CUL2* | 0.47 | VALIDATED |
| 25 | rs10758669 | 9 | 1.7E-09 | 2 | *JAK2* | 0.20 | VALIDATED |
| 26 | rs1551398 | 8 | 2.3E-09 | 2 | *NSMCE2* | 0.44 | VALIDATED |
| 27 | rs1456893 | 7 | 2.3E-09 | 4 | *FIGNL1* | 0.23 | VALIDATED |
| 28 | rs2872507 | 17 | 2.5E-09 | 17 | *IKZF3* | 0.23 | VALIDATED |
| 29 | rs1736135 | 21 | 3.7E-09 | 0 |  | N/A | VALIDATED |
| 30 | rs2476601 | 1 | 7.3E-09 | 8 | *PTPN22* | 0.0014 | VALIDATED |

Table S4B

| Region | SNP | Chr | *p_association_* | N (genes) | Implicated Gene | Text-Based *p*-value | Replication Study Result |
| --- | --- | --- | --- | --- | --- | --- | --- |
| 31 | rs4807569 | 19 | 1.1E-09 | 2 | *SBNO2* | 0.81 | INDETERMINATE |
| 32 | rs3763313 | 6 | 2.6E-09 | 8 | *BTNL2* | 0.23 | INDETERMINATE |
| 33 | rs10188217 | 2 | 6.4E-08 | 2 | *PUS10* | 0.46 | INDETERMINATE |
| 34 | rs12529198 | 6 | 3.5E-07 | 1 | *LYRM4* | 0.85 | INDETERMINATE |
| 35 | rs991804 | 17 | 5.3E-07 | 4 | CCL7 | 0.16 | INDETERMINATE |
| 36 | rs12985909 | 19 | 7.5E-07 | 2 | LSM4 | 0.64 | INDETERMINATE |
| 37 | rs780094 | 2 | 1.2E-06 | 22 | *SNX17* | 0.80 | INDETERMINATE |
| 38 | rs17309827 | 6 | 1.4E-06 | 2 | *SLC22A23* | 0.12 | INDETERMINATE |
| 39 | rs2738758 | 20 | 2.7E-06 | 10 | *TNFRSF6B* | 0.0038 | INDETERMINATE |
| 40 | rs707472 | 1 | 3.6E-06 | 4 | *UTS2* | 0.49 | INDETERMINATE |
| 41 | rs7758080 | 6 | 4.4E-06 | 4 | *SUMO4* | 0.033 | INDETERMINATE |
| 42 | rs13397985 | 2 | 9.0E-06 | 2 | *SP110* | 0.74 | INDETERMINATE |
| 43 | rs917997 | 2 | 1.1E-05 | 5 | *IL18RAP* | 0.0027 | INDETERMINATE |
| 44 | rs6128541 | 20 | 1.2E-05 | 1 | *EDN3* | 0.63 | INDETERMINATE |
| 45 | rs4253431 | 4 | 1.3E-05 | 3 | *CYP4V2* | 0.72 | INDETERMINATE |
| 46 | rs10863202 | 16 | 1.4E-05 | 4 | *IRF8* | 0.00058 | INDETERMINATE |
| 47 | rs8098673 | 18 | 1.4E-05 | 3 | *MIB1* | 0.18 | INDETERMINATE |
| 48 | rs7161377 | 14 | 2.3E-05 | 1 | *BATF* | 0.09 | INDETERMINATE |
| 49 | rs2688610 | 10 | 3.3E-05 | 10 | *CHCHD1* | 0.95 | INDETERMINATE |
| 50 | rs11758386 | 6 | 3.4E-05 | 2 | *CDKAL1* | 0.66 | INDETERMINATE |
| 51 | rs596308 | 11 | 3.5E-05 | 19 | *DNAJC4* | 0.76 | INDETERMINATE |
| 52 | rs2283790 | 22 | 4.6E-05 | 4 | *UBE2L3* | 0.30 | INDETERMINATE |

**Table S**4C

| Region | SNP | Chr | *p_association_* | N (genes) | Implicated Gene | Text-Based *p*-value | Replication Study Result |
| --- | --- | --- | --- | --- | --- | --- | --- |
| 53 | rs10010325 | 4 | 5.0E-05 | 1 | *TET2* | 0.99 | FAILED |
| 54 | rs10753415 | 1 | 5.1E-05 | 5 | *LIN9* | 0.73 | FAILED |
| 55 | rs11931489 | 4 | 5.3E-05 | 1 | *SORCS2* | 0.62 | FAILED |
| 56 | rs9829140 | 3 | 6.2E-05 | 5 | *CCRL1* | 0.46 | FAILED |
| 57 | rs6987445 | 8 | 1.3E-04 | 1 | *SNX16* | 0.43 | FAILED |
| 58 | rs2836757 | 21 | 1.7E-04 | 3 | *BRWD1* | 0.90 | FAILED |
| 59 | rs3011410 | 10 | 2.0E-04 | 1 | *BRWD2* | 0.13 | FAILED |
| 60 | rs6563216 | 13 | 2.2E-04 | 0 |  | N/A | FAILED |
| 61 | rs12827915 | 12 | 3.3E-04 | 4 | *KIAA1467* | 1.00 | FAILED |
| 62 | rs2944533 | 10 | 3.9E-04 | 1 | *TCERG1L* | 0.96 | FAILED |
| 63 | rs11959616 | 5 | 4.8E-04 | 2 | *WDR70* | 0.18 | FAILED |
| 64 | rs4543904 | 10 | 7.6E-04 | 1 | *ADARB2* | 0.52 | FAILED |
| 65 | rs1200610 | 1 | 9.3E-04 | 2 | *IVNS1ABP* | 0.58 | FAILED |
| 66 | rs7227145 | 18 | 9.5E-04 | 1 | *SERPINB5* | 0.38 | FAILED |
| 67 | rs17814449 | 7 | 9.6E-04 | 1 | *MKLN1* | 0.10 | FAILED |
| 68 | rs8111071 | 19 | 1.3E-03 | 10 | *FBXO46* | 0.54 | FAILED |
| 69 | rs9961822 | 18 | 1.8E-03 | 2 | *GRP* | 0.73 | FAILED |
| 70 | rs1040092 | 12 | 3.5E-03 | 2 | *LRIG3* | 0.71 | FAILED |
| 71 | rs158862 | 18 | 5.3E-03 | 1 | *NEDD4L* | 0.29 | FAILED |
| 72 | rs12607007 | 18 | 6.9E-03 | 4 | *TXNL4A* | 0.72 | FAILED |
| 73 | rs4073149 | 15 | 1.3E-02 | 16 | *UBL7* | 0.76 | FAILED |
| 74 | rs1784493 | 8 | 1.4E-02 | 2 | *OXR1* | 0.76 | FAILED |

**Table S4. Crohn’s Disease SNPs from a meta-analysis of GWA studies.** Here we list GRAIL results and summarize genotyping results for Crohn’s disease SNPs. These 74 SNPs emerged from a meta-analysis and as a result of replication genotyping, they were either validated (A), indeterminate (B), or failed (C). For each of the regions we list the SNP ID and the chromosome in the second and third column. In the fourth column we list the final combined association significance score of the SNP to the Crohn’s disease. In the fifth, sixth, and seventh columns we list GRAIL results including the number of genes in the region, the best candidate gene, and the text-based significance score for the region.
