# Supplementary material for: Identifying Relationships among Genomic Disease Regions: Predicting Genes at Pathogenic SNP Associations and Rare Deletions
Source: PLoS Genet. 2009 Jun 26;5(6):e1000534. doi: 10.1371/journal.pgen.1000534 (PMC2694358; doi:10.1371/journal.pgen.1000534)
Supplement: Table S5 — Algorithms to prioritize candidate genes. Our search of the literature identified nine algorithms that could be used to prioritize genes for replication. Four methods require no user-specified disease information (supervised), and five require some disease information from the user. We list in each row the name of the disease, the website, the necessary genetic data, the functional data used to prioritize genes, the disease-specific information that must be included, and the availability of the method. (0.09 MB DOC) [file pgen.1000534.s007.doc]

**TABLE S5**

|  | **Algorithm** | **Website** | **Genetic Input** | **Functional Information** | **Disease-specific Inputs** | **Availability** |
| --- | --- | --- | --- | --- | --- | --- |
| **Supervised** | ***Prioritizer*** | <http://humgen.med.uu.nl/~lude/prioritizer/> | All Associated Regions | GO terms, Protein Interaction Databases, Pathway Databases, and gene expression data | None | Java executable |
|  | ***Genes To Disease (G2D)*** | <http://www.ogic.ca/projects/g2d_2/> | Module 1,2 : 1 associated region  Module 3 : 2 genomic regions | Module 1 : MeSH and GO terms  Module 2 : GO terms; Module 3 : Protein-Protein Interactions | Module 1 : Phenotype (MIM number)  Module 2 : Disease genes  Module 3 : None | Online |
|  | ***Commonality of Functional Annotation (CFA)*** | <http://www.transvar.org/results/candi_gene/> | Multiple Associated Regions | GO terms | None | Python and R Scripts |
|  | ***Prospectr*** | <http://www.genetics.med.ed.ac.uk/prospectr/> | All associated genes | Sequence Characteristics | None | Online |
| **Unsupervised** | ***Endeavour*** | <http://homes.esat.kuleuven.be/~bioiuser/endeavour/endeavourweb.php> | Candidate genes | Functional databases (annotation, expression, interaction, BLAST) | Disease genes | Online |
|  | ***GeneSeeker*** | <http://www.cmbi.ru.nl/GeneSeeker/basic_form.html> | 1 associated region | Expression and Phenotype Databases | Tissue or Phenotype description | Online |
|  | ***SUSPECTS*** | [http://www.genetics.med.ed.ac.uk/suspects/](http://www-micrel.deis.unibo.it/~tom/) | 1 associated region | GO terms, protein domains and expression data | Disease genes, phenotype | Online |
|  | ***TOM*** | http://www-micrel.deis.unibo.it/~tom/ | Module 1 : 1 associated region  Module 2 : 2 associated regions | Mapping, expression and functional databases | Disease genes | Online |
|  | ***CANDID*** | <https://dsgweb.wustl.edu/hutz/candid.html> | linkage/  association file | Literature, protein-domains, conservation information, tissue expression, and protein interactions | Keywords, tissue, connectivity data | Online |

**Table S5. Algorithms to prioritize candidate genes.** Our search of the literature identified nine algorithms that could be used to prioritize genes for replication. Four methods require no user-specified disease information (supervised), and five require some disease information from the user. We list in each row the name of the disease, the website, the necessary genetic data, the functional data used to prioritize genes, the disease-specific information that must be included, and the availability of the method.
