# Supplementary material for: Identifying Relationships among Genomic Disease Regions: Predicting Genes at Pathogenic SNP Associations and Rare Deletions
Source: PLoS Genet. 2009 Jun 26;5(6):e1000534. doi: 10.1371/journal.pgen.1000534 (PMC2694358; doi:10.1371/journal.pgen.1000534)
Supplement: Table S6 — Performance measures for prioritization algorithms. We used five algorithms (column 1) to score putatively associated SNPs from the Crohn's meta-analysis. After calculating an ROC curve for each algorithm, we calculated the AUC (column 2). We also calculated a p-value with a one-tailed rank-sum test comparing the median rank of the validated SNPs to the median rank of the failed SNPs (column 2). (0.04 MB DOC) [file pgen.1000534.s008.doc]

**TABLE S6**

| Prioritization Algorithm | Area Under the Curve | Validated vs Failed SNPs (*p*) |
| --- | --- | --- |
| ***GRAIL*** | 0.75 | 0.00064 |
| ***Prioritizer*** | 0.60 | 0.11 |
| ***G2D*** | 0.61 | 0.081 |
| ***CFA*** | 0.57 | 0.13 |
| ***PROSPECTR*** | 0.54 | 0.38 |

**Table S6. Performance measures for prioritization algorithms.** We used five algorithms (column 1) to score putatively associated SNPs from the Crohn’s meta-analysis. After calculating an ROC curve for each algorithm, we calculated the AUC (column 2). We also calculated a *p*-value with a one-tailed rank-sum test comparing the median rank of the validated SNPs to the median rank of the failed SNPs (column 2).
