# Supplementary material for: Identifying Relationships among Genomic Disease Regions: Predicting Genes at Pathogenic SNP Associations and Rare Deletions
Source: PLoS Genet. 2009 Jun 26;5(6):e1000534. doi: 10.1371/journal.pgen.1000534 (PMC2694358; doi:10.1371/journal.pgen.1000534)
Supplement: Table S7 — Other promising regions in Crohn's Disease GWA meta-analysis. Information about the top six regions identified by GRAIL from the next 75 most significant regions from the Crohn's GWA study. All associations are indeterminate, and association p-values are taken from the GWA meta-analysis - these regions have not yet been replicated. (0.05 MB DOC) [file pgen.1000534.s009.doc]

**TABLE S7**

| SNP | Chr | *p_association_* | N (genes) | Implicated Gene | Text-Based *p*-value | Replication Study Result |
| --- | --- | --- | --- | --- | --- | --- |
| rs8178556 | 21 | 6.6E-05 | 2 | *IFNAR1* | 0.00017 | INDETERMINATE |
| rs12928822 | 16 | 1.1E-04 | 6 | *SOCS1* | 0.00082 | INDETERMINATE |
| rs9594759 | 13 | 1.5E-04 | 3 | *TNFSF11* | 0.0011 | INDETERMINATE |
| rs10509566 | 10 | 9.8E-05 | 11 | *FAS* | 0.0013 | INDETERMINATE |
| rs2853705 | 3 | 1.0E-04 | 2 | *CCR8* | 0.0064 | INDETERMINATE |
| rs7753394 | 6 | 5.5E-05 | 2 | *TNFAIP3* | 0.0075 | INDETERMINATE |

**Table S7. Other promising regions in Crohn’s Disease GWA meta-analysis.** Information about the top six regions identified by GRAIL from the next 75 most significant regions from the Crohn’s GWA study. All associations are indeterminate, and association *p*-values are taken from the GWA meta-analysis – these regions have not yet been replicated.
