# Supplementary material for: Identifying Relationships among Genomic Disease Regions: Predicting Genes at Pathogenic SNP Associations and Rare Deletions
Source: PLoS Genet. 2009 Jun 26;5(6):e1000534. doi: 10.1371/journal.pgen.1000534 (PMC2694358; doi:10.1371/journal.pgen.1000534)
Supplement: Table S8 — Rare or de novo schizophrenia control deletions. Here we list all of the deletions that GRAIL identified as most related to other deleted genes (ptext<0.05). For each deletion we list the chromosome, the range of the deletion, the GRAIL p-value for the region, and the best candidate gene in the region identified by GRAIL. Most genomic coordinates are listed in HG17. * HG18 coordinates. (0.06 MB DOC) [file pgen.1000534.s010.doc]

**TABLE S8**

| **CHR** | **Start** | **Stop** | ***p_text_*** | **Candidate Gene** |
| --- | --- | --- | --- | --- |
| 12 | 128,541,753 | 128,777,246 | 0.0022 | *TMEM132D* |
| 6 | 130,174,633 | 130,470,447 | 0.0023 | *L3MBTL3* |
| 9 | 20,791,991 | 20,935,372 | 0.0028 | *KIAA1797* |
| 6 | 12,888,976 | 12,990,050 | 0.0042 | *PHACTR1* |
| 10 | 134,028,972 | 134,163,326 | 0.015 | *LRRC27* |
| 11 | *31,163,933 | 31,272,108 | 0.019 | *DCDC1* |
| 1 | 16,590,238 | 16,732,157 | 0.025 | *NBPF1* |
| 1 | 171,281,334 | 171,422,374 | 0.027 | *RABGAP1L* |
| 9 | 72,320,846 | 72,468,136 | 0.044 | *TMC1* |
| 10 | 117,331,154 | 117,439,540 | 0.048 | *ATRNL1* |
| 15 | 25,794,860 | 25,976,300 | 0.049 | *OCA2* |

**Table S8.** **Rare or de novo schizophrenia control deletions.** Here we list all of the deletions that GRAIL identified as most related to other deleted genes (*p_text_* < 0.05). For each deletion we list the chromosome, the range of the deletion, the GRAIL p-value for the region, and the best candidate gene in the region identified by GRAIL. Most genomic coordinates are listed in HG17. * HG18 coordinates.
