# Supplementary material for: Identifying Relationships among Genomic Disease Regions: Predicting Genes at Pathogenic SNP Associations and Rare Deletions
Source: PLoS Genet. 2009 Jun 26;5(6):e1000534. doi: 10.1371/journal.pgen.1000534 (PMC2694358; doi:10.1371/journal.pgen.1000534)
Supplement: Text S1 — A. Random SNP groups; B. Comparison of GRAIL to other related algorithms. (0.09 MB DOC) [file pgen.1000534.s011.doc]

**Text S1**

1. **Random SNP groups**

Here we tested GRAIL by scoring random groups of SNPs, and characterized the resulting *p_text_* values to insure that they accurately estimate type I error and approximate true *p*-values.

We defined 100 groups of 50 random SNPs. In order to mimic a GWA study as closely as possible, we selected each of the SNPs at random from a set of SNPs on the Affymetrix 500 K SNP array that passed quality assurance in a separate rheumatoid arthritis meta-analysis [1]. We applied GRAIL to each random group to score SNPs. We noted the *p_text_* for each of the 50 SNP and calculated the percentage of SNPs that GRAIL assigned *p_text_* scores that were less than 0.0001, 0.001, 0.01, 0.1, 0.25, and 0.5. If *p_text_* scores approximate type I error appropriately, the fraction of SNPs in a random group scoring below a threshold should be approximately equal to the threshold, since all of these SNPs are false positives.

In **Figure S1** we present this data. Overall, *p_text_* thresholds correlate well with SNP fractions. For example, only a median of 7% of SNPs per group score *p_text_* < 0.05, and only a median of 2% of SNPs per group score *p_text_ <* 0.01. This does suggest that some modest inflation. Random SNP groups rarely have individual SNPs that score *p_text_* < 0.001.

**B. Comparison of GRAIL to other related algorithms**

We conducted a broad search to find related algorithms that prioritize candidate genes in putatively associated genomic regions; we required algorithms to have publicly available implementations. We identified nine related algorithms; all assign candidate genes a priority score that suggests the likelihood that it is a true disease gene (see **Table S5**). We found two types of tools: (1) *unsupervised* tools that require no disease-specific hypotheses and (2) *supervised* tools that require user-specified disease hypotheses, such as known genes or keywords describing relevant pathogenic pathways. There were four unsupervised tools: ***Prioritizer*** [2], ***Gene2Disease*** (***G2D***) [3,4,5], ***Commonality of Functional Annotation*** (***CFA***) [6], and ***Prospectr*** [7]. There were five supervised tools: ***Endeavour*** [8], ***GeneSeeker*** [9], ***SUSPECTS*** [10], ***TOM*** [11], and ***CANDID*** [12].

We sought to compare GRAIL against related algorithms in its ability to predict which of the 74 putatively associated Crohn’s SNPs represent true disease associations [13]. This is the exact same test, described in the main text (see **Results** section, **Methods** section and **Figure 3**), that we applied to GRAIL. For many complex diseases, and in particular Crohn’s disease, there are no definitive hypotheses about the predisposing genetic mechanisms; thus supervised algorithms were difficult to objectively evaluate in this instance. We therefore tested only the unsupervised tools and excluded the supervised tools from our analysis. For each of these algorithms, we scored the 74 putative SNP associations and assessed their ability to prioritize true disease genes. Since most methods require associated genomic regions, we converted Crohn’s SNPs into associated SNP regions based on LD properties using the same strategy employed in the first step of GRAIL (see **Methods** and **Figure 1**).

Prioritizer integrates gene annotations, pathway databases, protein interaction data, and gene expression data into a gene network; for each gene it returns a *p*-value that represents the significance of its connectivity to genes in other disease regions which hypothetically represents its likelihood to play a role in disease. As input it requires multiple disease regions. We ran all 74 associated SNP regions together and obtained the *p-*values assigned by Prioritizer. We assigned each SNP region the best *p*-value from its most significant gene, after a Bonferroni correction for the number of genes within the region. Bonferroni correction resulted in improved performance (data not shown).

We used G2D’s first module, which scores genes within an individual genomic region based on relationships derived from disease symptoms (MeSH C terms) and features of genes identified in the region (GO terms). For input it requires a relevant OMIM number and a single disease region. As suggested by the authors for complex traits with multiple associated loci [4], we ran each of the 74 disease regions independently using the OMIM identifier 266600 (Crohn’s Disease). G2D assigns an *R*-score to each gene in a disease region; values close to zero indicate a possible relationship to disease (see website). We assigned each disease region the lowest *R*-score of genes within it. Other G2D modules were not applicable to our evaluation; module 2 required a set of known disease genes (supervised) and module 3 could only test genes within two regions against each other.

CFA tests candidate gene sets for enrichment of Gene Ontology (GO) terms [6]. It scores and ranks genes that are enriched for shared GO terms, with the assumption that genes that play a role in disease most likely are in a common pathway. We assigned each associated SNP region the best CFA score of genes within that region.

Prospectr scores genes based on sequence-based features, which the authors hypothesize indicate likelihood to be involved in disease [7]. We identified overlapping genes for each SNP region. We then assigned each region the best Prospectr score of genes within that region.

After scoring 74 SNP regions with each of the four algorithms, we calculated sensitivity and specificity using only validated and failed SNPs at a range of cutoff levels. SNPs that obtain intermediate significance in follow-up genotyping were excluded. We used that data to generate an ROC curve (**Figure** **S3)** and area under the curve (AUC, **Table S6**). We also calculated a one-tailed non-parametric rank-sum statistic comparing the median scores of validated versus failed SNPs.

For each of the tested metrics, GRAIL demonstrates superior performance to other tested algorithms. GRAIL demonstrates greater sensitivity than all other methods consistently across a broad range of levels of specificity. GRAIL has an area under the curve (AUC) in excess of each of the other algorithms. Finally, GRAIL is able to separate failed and validated SNPs more effectively than other algorithms. Other algorithms demonstrate nominally significant separations between validated and failed SNPs (*p*>0.08).

**REFERENCES**

1. Raychaudhuri S, Remmers EF, Lee AT, Hackett R, Guiducci C, et al. (2008) Common variants at CD40 and other loci confer risk of rheumatoid arthritis. Nat Genet 40: 1216-1223.

2. Franke L, van Bakel H, Fokkens L, de Jong ED, Egmont-Petersen M, et al. (2006) Reconstruction of a functional human gene network, with an application for prioritizing positional candidate genes. Am J Hum Genet 78: 1011-1025.

3. Perez-Iratxeta C, Bork P, Andrade-Navarro MA (2007) Update of the G2D tool for prioritization of gene candidates to inherited diseases. Nucleic Acids Res 35: W212-216.

4. Perez-Iratxeta C, Wjst M, Bork P, Andrade MA (2005) G2D: a tool for mining genes associated with disease. BMC Genet 6: 45.

5. Tremblay K, Lemire M, Potvin C, Tremblay A, Hunninghake GM, et al. (2008) Genes to diseases (G2D) computational method to identify asthma candidate genes. PLoS ONE 3: e2907.

6. Shriner D, Baye TM, Padilla MA, Zhang S, Vaughan LK, et al. (2008) Commonality of functional annotation: a method for prioritization of candidate genes from genome-wide linkage studies. Nucleic Acids Res 36: e26.

7. Adie EA, Adams RR, Evans KL, Porteous DJ, Pickard BS (2005) Speeding disease gene discovery by sequence based candidate prioritization. BMC Bioinformatics 6: 55.

8. Tranchevent LC, Barriot R, Yu S, Van Vooren S, Van Loo P, et al. (2008) ENDEAVOUR update: a web resource for gene prioritization in multiple species. Nucleic Acids Res 36: W377-384.

9. van Driel MA, Cuelenaere K, Kemmeren PP, Leunissen JA, Brunner HG, et al. (2005) GeneSeeker: extraction and integration of human disease-related information from web-based genetic databases. Nucleic Acids Res 33: W758-761.

10. Adie EA, Adams RR, Evans KL, Porteous DJ, Pickard BS (2006) SUSPECTS: enabling fast and effective prioritization of positional candidates. Bioinformatics 22: 773-774.

11. Rossi S, Masotti D, Nardini C, Bonora E, Romeo G, et al. (2006) TOM: a web-based integrated approach for identification of candidate disease genes. Nucleic Acids Res 34: W285-292.

12. Hutz JE, Kraja AT, McLeod HL, Province MA (2008) CANDID: a flexible method for prioritizing candidate genes for complex human traits. Genet Epidemiol.

13. Barrett JC, Hansoul S, Nicolae DL, Cho JH, Duerr RH, et al. (2008) Genome-wide association defines more than 30 distinct susceptibility loci for Crohn's disease. Nat Genet 40: 955-962.
